# Supplementary material for: How well are marginalised groups represented in electronic records? A codelist development project and cross-sectional analysis of UK electronic health records
Source: BMJ Open. 2025 Aug 11;15(8):e098305. doi: 10.1136/bmjopen-2024-098305 (PMC12359504; doi:10.1136/bmjopen-2024-098305)
Supplement: Author's manuscript [file bmjopen-2024-098305.draft_revisions.pdf]

BMJ Open is committed to open peer review. As part of this commitment we make the peer review history of every article we publish publicly available.

When an article is published we post the peer reviewers' comments and the authors' responses online. We also post the versions of the paper that were used during peer review. These are the versions that the peer review comments apply to.

The versions of the paper that follow are the versions that were submitted during the peer review process. They are not the versions of record or the final published versions. They should not be cited or distributed as the published version of this manuscript.

BMJ Open is an open access journal and the full, final, typeset and author-corrected version of record of the manuscript is available on our site with no access controls, subscription charges or pay-per-view fees (http://bmjopen.bmj.com).

If you have any questions on BMJ Open's open peer review process please email info.bmjopen@bmj.com

BMJ Open

# Development and evaluation of codelists for identifying marginalised groups in primary care.



| | |
|---|---|
| Journal: | *BMJ Open* |
| Manuscript ID | bmjopen-2024-098305 |
| Article Type: | Original research |
| Date Submitted by the Author: | 20-Dec-2024 |





| | |
|---|---|
| Complete List of Authors: | Perchyk, Tetyana; University of Surrey, School of Health Sciences<br>de Vere Hunt, Isabella; University of Oxford, Nuffield Department of Primary Care Health Sciences<br>Nicholson, Brian; University of Oxford, Nuffield Department of Primary Care Health Sciences<br>Mounce, Luke; University of Exeter, Medical School<br>Sykes, Kate; Northumbria University, Faculty of Health and Lide Sciences<br>Lyratzopoulos, Georgios ; University College London, Department of Behavioural Science and Health<br>Lemanska, Agnieszka; University of Surrey; National Physical Laboratory, Data Science Department<br>Whitaker, Katriina; University of Surrey, School of Health Sciences<br>Kerrison, Robert; University of Surrey, School of Health Sciences |





| | |
|---|---|
| Keywords: | Primary Care < Primary Health Care, EPIDEMIOLOGY, MENTAL HEALTH, Transgender Persons |



SCHOLARONE™ Manuscripts

*I, the Submitting Author has the right to grant and does grant on behalf of all authors of the Work (as defined in the below author licence), an exclusive licence and/or a non-exclusive licence for contributions from authors who are: i) UK Crown employees; ii) where BMJ has agreed a CC-BY licence shall apply, and/or iii) in accordance with the terms applicable for US Federal Government officers or employees acting as part of their official duties; on a worldwide, perpetual, irrevocable, royalty-free basis to BMJ Publishing Group Ltd ("BMJ") its licensees and where the relevant Journal is co-owned by BMJ to the co-owners of the Journal, to publish the Work in this journal and any other BMJ products and to exploit all rights, as set out in our licence.*

*The Submitting Author accepts and understands that any supply made under these terms is made by BMJ to the Submitting Author unless you are acting as an employee on behalf of your employer or a postgraduate student of an affiliated institution which is paying any applicable article publishing charge ("APC") for Open Access articles. Where the Submitting Author wishes to make the Work available on an Open Access basis (and intends to pay the relevant APC), the terms of reuse of such Open Access shall be governed by a Creative Commons licence – details of these licences and which Creative Commons licence will apply to this Work are set out in our licence referred to above.*

*Other than as permitted in any relevant BMJ Author's Self Archiving Policies, I confirm this Work has not been accepted for publication elsewhere, is not being considered for publication elsewhere and does not duplicate material already published. I confirm all authors consent to publication of this Work and authorise the granting of this licence.*

For peer review only

**TITLE.** Development and evaluation of codelists for identifying marginalised groups in primary care.

**Corresponding author.** Tetyana Perchyk[1] (ORCID: 0009-0002-0273-8087)

**Co-authors.** Isabella de Vere Hunt[2] (ORCID: 0000-0002-4238-0924), Brian D Nicholson[2] (ORCID: 0000-0003-0661-7362), Luke Mounce[3] (ORCID: 0000-0002-6089-0661), Kate Sykes[4] (ORCID: 0000-0002-2942-7193), Yoryos Lyratzopoulos[5] (ORCID: 0000-0002-2873-7421), Agnieszka Lemanska[1,6] (ORCID: 0000-0003-4849-2430)[1], Katriina L Whitaker[1] (ORCID: 0000-0002-0947-1840) and Robert S Kerrison[1] (ORCID: 0000-0002-8900-749X)

**Affiliations.** [1]University of Surrey, School of Health Sciences, United Kingdom; [2]University of Oxford, Nuffield Department of Primary Care Health Sciences, United Kingdom; [3]University of Exeter, Medical School, United Kingdom; [4]Northumbria University, Faculty of Health and Life Sciences, United Kingdom; [5]UCL, Department of Behavioural Science and Health, United Kingdom; [6]National Physical Laboratory, Data Science Department, United Kingdom.

**Funding.** This work is funded by the National Institute for Health Research (NIHR) Policy Research Programme, conducted through the Policy Research Unit in Cancer Awareness, Screening and Early Diagnosis, NIHR206132. The work of NPL co-authors was partly funded by the UK Government's Department for Science, Innovation & Technology through the UK's National Measurement System programmes via the Semantic Technologies theme. The views expressed are those of the author(s) and not necessarily those of the NIHR or the Department of Health and Social Care.

This work was supported by Breast Cancer Now [Grant Ref no: 2023FebIFS1615].

**Ethical approval.** This project did not involve the collection of primary data. As such, ethical approval was not required.

**Data availability.** The codelists developed through this work are available from Open Science Framework: https://osf.io/8skze/

**Competing interests.** The authors have no conflicts of interest to declare.

**ABSTRACT.**

**Objectives.** Primary care electronic health records provide a rich source of information for inequalities research. However, the reliability and validity of the research derived from these records depends on the completeness and resolution of the codelists used to identify populations of interest. The aim of this project was to develop comprehensive codelists for identifying people from ethnic minority groups, people with learning disabilities (LD), people with severe mental illness (SMI) and people who are transgender.

**Design.** We followed a three-stage process to define and extract relevant codelists. First, groups of interest were defined a priori. Next, relevant clinical codes, relating to the groups, were identified by searching Clinical Practice Research Datalink (CPRD) publications, codelist repositories and the CPRD code browser. Relevant codelists were extracted and merged according to group, and duplicates were removed. Finally, remaining codes were reviewed by two general practitioners.

**Setting.** The curated codelists were compared using a representative sample in the United Kingdom. The frequencies of individuals identified using the curated codelists were assessed and compared to widely used alternative codelists.

**Participants.** Comprehensiveness was assessed in a representative CPRD population of 10,966,759 people.

**Results.** A total of 52 codelists were identified. From these, 1,420 unique codes were selected after removal of duplicates and GP review. Compared with comparator codelists, an additional 48,017 (76.6%), 52,953 (68.9%) and 508 (36.9%) people with a LD, SMI or transgender code were identified. The frequencies identified for ethnicity were consistent with expectations for the UK population.

**Conclusions.** The curated codelists are more sensitive than those widely used in practice and research. The codelists curated through this project will improve inequalities research by improving standards of identifying marginalised groups in primary care data.

**STRENGTHS AND LIMITATIONS.**

- This study used replicable methods to develop comprehensive codelists for the identification of groups known to experience healthcare inequalities.
- A systematic approach was used with a priori identification of the clinical features and a Delphi review of identified codes.
- The codelists were validated and assessed in a representative sample.
- The codelists were made available for use both nationally and internationally; however, additional review is recommended to ensure they fit research-specific questions.



For peer review only - http://bmjopen.bmj.com/site/about/guidelines.xhtml

## MAIN TEXT

### 1. INTRODUCTION.

Health inequalities are unfair and avoidable differences in health, which are systematically established and arise from the social conditions in which people are born, grow, live, work and age [1].

In the United Kingdom (UK), health inequalities are a national priority [2]. The National Health Service for England (NHS England) developed 'The CORE20Plus5 Framework', which outlines priority groups and clinical areas requiring accelerated improvement [3]. The 'Core20' refers to the 20% most deprived areas in England, while the 'Plus' refers to population groups that should be identified at a local level, including (but not limited to): ethnic minority groups, people with a learning disability (LD) or autism, people with multiple long-term health conditions, other groups that share protected characteristics (as defined by the Equality Act 2010 [4]), and groups experiencing social exclusion (such as coastal communities, where there may be small areas of high deprivation hidden amongst relative affluence) [3]. The '5' describes key clinical areas requiring accelerated improvement for these populations, namely: maternity, severe mental illness (SMI), chronic respiratory disease, early cancer diagnosis and hypertension case-finding and management [3].

While the CORE20Plus5 framework provides a useful outline of national priorities for health research and clinical practice, there are several ongoing challenges that need to be addressed for researchers to support national strategy [5]. Pertinent among these is the identification and classification of CORE20Plus groups (also referred to as 'socially disadvantaged' or 'marginalised' groups) within electronic health records (EHRs) [5]. EHRs include key administrative clinical data relevant to a person's care, including demographics, progress notes, problems, medications, vital signs, past medical history, immunisations, laboratory data and radiology reports [6]. These data are recorded using clinical coding systems, typically containing hundreds of thousands of concepts [7], which need to be categorised via codelists to facilitate research.

While methods for codelist development and reporting have previously been published [8–10], and many studies now report the methods used to develop codelists for various medical diagnoses, few papers describe the systematic development of codelists specifically for identifying marginalised populations [9]. Some papers have attempted to develop codelists for certain marginalised groups, including for identifying the autistic population [11], the LGB population [12], and for people who experience multiple long-term conditions [13]. However, there does not appear to be any papers describing the development of codelists for other marginalised groups.

To support future health inequalities research, we set out to develop comprehensive codelists for the identification of four CORE20plus5 groups, namely: people from ethnic minority groups, people with



For peer review only - http://bmjopen.bmj.com/site/about/guidelines.xhtml

LDs, people with SMI and people who are transgender. We selected these groups because there is a general lack of standardised methods for codelist development to describe these inequality populations in EHRs.

## 2. METHOD.

Previously published approaches for codelist development were reviewed [9,10], and Watson et al's [14] approach was deemed the most appropriate because it specifically sets out methods for developing codelists using UK based EHRs.

The approach comprises three steps: **Step 1.** Clearly define the clinical feature of interest a priori; **Step 2.** Assemble a list of codes that may be used to record the clinical feature; **Step 3.** Delphi review of codes.

The following presents a detailed overview of the processes undertaken for each step.

### 2.1. Step 1. Clearly define the clinical feature of interest a priori.

Watson et al recommend beginning by clearly defining the clinical feature of interest [14]. To do this, they recommend using reliable sources of information, such as the National Institute for Health and Care Excellence (NICE) [15]. For the purposes of this study, we used the following definitions and sources to define marginalised groups of interest:

**Ethnicity.** The Office of National Statistics (ONS) [16] has highlighted that there is no true consensus on what defines an ethnic group, as identification to these are "self-defined and subjectively meaningful to the individual". However, as stated by NHS England, it is generally accepted that ethnicity includes a variety of elements, such as ancestry, culture, identity, religion, language, and physical appearance [17].

We defined ethnicity according to the characteristics set out in the Equality Act 2010, namely: colour, nationality and ethnic or national origins [4]. To this end, UK Census 2021 categories were used to group ethnicity codes (see Table 1) [18].

| Table 1. UK census ethnic categories [15]. |
|---|
| **Asian or Asian British** |
| Indian |
| Pakistani |
| Bangladeshi |
| Chinese |
| Any other Asian background |
| **Black, Black British, Caribbean or African** |
| Caribbean |
| African |
| Any other Black, Black British, or Caribbean background |
| **Mixed or multiple ethnic groups** |
| White and Black Caribbean |
| White and Black African |
| White and Asian |
| Any other Mixed or multiple ethnic background |
| **White** |
| English, Welsh, Scottish, Northern Irish or British |
| Irish |
| Gypsy or Irish Traveller |
| Roma |
| Any other White background |
| **Other ethnic group** |
| Arab |
| Any other ethnic group |



For peer review only - http://bmjopen.bmj.com/site/about/guidelines.xhtml

**Learning disability.** The Department of Health and Social Care (DHSC) [19] defines a learning disability as: “a significantly reduced ability to understand new or complex information, to learn new skills (impaired intelligence), with a reduced ability to cope independently (impaired social functioning), which started before adulthood.” According to Public Health England [20]: “a learning disability is different to a learning difficulty, which is a reduced intellectual ability for a specific form of learning and includes conditions such as dyslexia (reading), dyspraxia (affecting physical co-ordination) and attention deficit hyperactivity disorder (ADHD).”

We defined learning disabilities according to the characteristics described by the DHSC. We did not categorise learning disabilities into groups. They are often described as being ‘mild’, ‘moderate’, or ‘severe’, ‘verbal’ or ‘non-verbal’, and ‘syndromic’ or ‘non-syndromic’, but there is no consensus as to which learning disabilities fall into which categories, and there is individual-level variation within the same disability, making it inappropriate to generalise [21].

**Severe mental illness.** The Diagnostic and Statistical Manual, fifth edition (DSM-V), defines severe mental illness as: “a mental, behavioural, or emotional disorder resulting in serious functional impairment, which substantially interferes with or limits one or more major life activities.” [22].

We defined SMI according to the characteristics described in the DSM-V, and categorised codes according to four recognised conditions, namely: ‘schizophrenia’, ‘bipolar disorder’, ‘severe major depression’ and ‘other psychotic disorders’ [22].

**Transgender.** The UK Government Equality Office [23] defines trans (transgender) people as “People whose gender is different from the gender assigned to them at birth.” They provide the following by way of example: “A trans man is someone that transitioned from woman to man” [23].

We used the definition provided by the Government Equality Office [23]. Most clinical codes do not differentiate between trans men and trans women; therefore, we did not group codes according to the direction of the transition (i.e. male to female; female to male).

**Public involvement.**

Members of the public who experience one or more of the inequalities of interest were involved in the design of this study. They aided our understanding of how to properly define these inequality groups and gave insight into their experiences with primary care.

**2.2. Step 2.** Assemble list of codes that may be used to record the clinical feature.

In the second step, an online thesaurus was used to develop a list of synonyms associated with the clinical features of interest. An overview of the synonyms used for each clinical feature is presented in Table 2.



For peer review only - http://bmjopen.bmj.com/site/about/guidelines.xhtml

| Table 2. List of synonyms used for each clinical feature | |
|---|---|
| **Clinical feature** | **Synonyms** |
| **Ethnicity** | Nationality, Nationalities, Nation(s), Race(s), Racial, Colour, Ethnic, Ethnicity, Minority, Minorities |
| **Learning Disability** | Intellectual disability, Intellectual disabilities, Learning disability, Learning Disabilities, ID(s), LD(s) |
| **Severe mental illness** | Serious mental illness, Serious mental illnesses, Severe mental illness, Severe mental illnesses, SMI(s) |
| **Transgender** | Trans, Transsexual, Transgender, Transgender, Gender dysmorphia, Trans man, Trans woman, Trans men, Trans women, Transfeminine, Transmasculine, Gender Surgery, LGBT. |

We then searched for published codelists, relating to the clinical features, using the synonyms identified. One major resource used was the October 2023 Clinical Practice Research Datalink (CPRD) Bibliography [24]. CPRD is one of the largest research databases of electronic, anonymised, and longitudinal medical records from primary care in the world [25]. The CPRD Bibliography is a resource which provides a record of all published studies (>3,000 as of October 2023) using CPRD data, and this aided in our search for relevant publications. The codelists used in these publications were subsequently downloaded, where they had been made available.

To capture codelists used in non-CPRD studies, we also searched for, and downloaded, relevant codelists from four widely used codelist repositories, namely: OpenCodelists [26], the Health Data Research UK (HDRUK) Phenotype Library [27], the University of Cambridge Codelist Repository [28] and the London School of Hygiene and Tropical Medicine (LSHTM) Data Compass [29].

The codes from the codelists were then extracted and collated into separate excel files for ethnicity, LD, SMI and transgender. Duplicate codes were removed, and remaining codes grouped by the two main coding systems used in the UK: Read and systematized nomenclature of medicine clinical terminology (SNOMED-CT). Read was generally used in primary care until 2018, and SNOMED-CT thereafter, following the enablement of SNOMED-CT within Primary care systems [30].

The aggregated codelists were then checked for accuracy using the CPRD code browser, which contains the diagnostic description, the alphanumeric Read / SNOMED-CT code originally used by the general practitioner (GP) to enter the clinical information, and CPRD's proprietary 'medcode' / 'medcodeid' (which is simply a numeric equivalent of the Read / SNOMED-CT code) [30]. The tool allowed for easy browsing by either using a known code or description to check code accuracy. In line with the method described by Watson et al [14], inaccurate codes were removed or corrected, and additional codes (not previously identified) added if they were associated with the diagnostic definition of one or more eligible codes.



For peer review only - http://bmjopen.bmj.com/site/about/guidelines.xhtml

### 2.3. **Step 3.** Delphi review of codes.

In the final step, the codelists were sent to a GP (IVH) for review. They scored each code using the following three-point scale:

1. Definitely include: the code accurately defines the clinical feature of interest, and GPs would use it.
2. Uncertain: it remains unclear whether the code accurately reflects the clinical feature of interest, or whether GPs would use it.
3. Definitely exclude: the code does not define the clinical feature of interest, and GPs would avoid.

Codes assigned a score of 2 (‘Uncertain’) or 3 (‘Definitely exclude’) were reviewed by a second GP (BN). All codes assigned a score of 3 (‘Definitely exclude’) by both reviewers were removed from the codelists. All codes receiving a score of 2 from either reviewer were retained as ‘uncertainty variables’, which are recommended for sensitivity analyses [14]. These variables are typically less frequently used by GPs, but may identify additional, potentially relevant, cases [14].

### 2.4. Assessing codelist comprehensiveness

To explore the comprehensiveness of the developed codelists, we used the CPRD database browser to ascertain the number of people with at least one recording of an included code for each list. We restricted the population to those who had been registered with their CPRD Aurum practice since at least 1st January 2019 and who had neither died nor deregistered from their practice by August 2022 (the date of last data collection).

In the context of CPRD studies, researchers have been able to acquire already categorised ethnicity data through linkage with the Hospital Episode Statistic (HES) [31]. Derived ethnicity data is also now available through CPRD at an additional cost [32]. The ethnicity codelist we generated will service to those researchers who are using other non-CPRD datasets, or ones who may not have access to either the linked HES data or the CPRD derived ethnicity.

For the other variables, validated codelists from other sources were selected as comparisons, seeking to match the scope of each list as closely as possible. Codelists for LD and SMI were sourced from the Quality and Outcomes Framework (QOF: the pay for performance scheme for general practices in the UK) [33]. QOF includes incentives for practices to keep a register of patients with certain conditions, including LD and SMI, and produces validated codelists for this purpose. We used lists from the QOF Business Rules version 45; “Learning Disability codes” (list name LD_COD) and "Psychosis and schizophrenia and bipolar affective disease codes" (list MH_COD) as our comparison lists. For the transgender category, no QOF list exists, and literature is slim. We identified two identical previously published codelists that were used to compare to our newly developed codelist.



For peer review only - http://bmjopen.bmj.com/site/about/guidelines.xhtml

## 3. RESULTS.

A review of 72 relevant publications and additional codelist repositories identified a total of 52 codelists: 16 for ethnicity, 16 for LD, 18 for SMI and 2 for transgender. After removing duplicates and Delphi review, 379 codes were excluded, and 1,420 total unique codes remained: 325 codes for ethnicity, 558 codes for LD, 499 codes for SMI and 38 codes for transgender. The full review process is demonstrated in

(Figure 1 Here)

The codes were run in the source population of CPRD (n=10,966,759) to ascertain the number of people captured by the codelists (see Table 3). People were identified by the presence of one or more codes related to the inequality group of interest. 110,692 people were found to have LD (1.00%), 129,788 people had SMI (1.18%) and 1,884 people were transgender (0.02%). This represented an increased capture of 48,017 (76.6%) for LD, 52,953 (68.9%) for SMI, and 508 (36.9%) for transgender (see Table 3).

Ethnicity data were available for 4,384,017 (40.0%) individuals. Of these, 3,112,319 (71.0%) were ethnically white; 285,126 (6.50%) were ethnically Asian; 116,570 (2.66%) were ethnically black; 63,024 (1.44%) were of mixed ethnicity; 94,693 (2.16%) were categorised as ‘other;’ and 712,286 (16.3%) were of unknown ethnicity (see table 3).

For peer review only



For peer review only - http://bmjopen.bmj.com/site/about/guidelines.xhtml

**Table 3.** Comparing frequencies derived from original and curated codelist for LD, SMI and transgender as well as frequencies between ethnic groups.

| Total population (N) | Mean age (SD*) | Age range | Frequency of males (%) | Frequency of females (%) |
|---|---|---|---|---|
| 10,966,759 | 43.3 (22.8) | 4 – 100 years old | 5,554,024 (50.6) | 5,412,735 (49.4) |

| Inequality Group | Original Codelist (N) | Curated Codelist (N) | Difference in Identification (N) | Percent Increase (%) |
|---|---|---|---|---|
| **Learning disability** | 62,675 | 110,692 | 48,017 | 76.6 |
| **Severe mental illness** | 76,835 | 129,788 | 52,953 | 68.9 |
| **Transgender** | 1,376 | 1,884 | 508 | 36.9 |

| Ethnicity | Frequency from curated codelist (N) | Frequency percent (%) |
|---|---|---|
| **White** | 3,112,319 | 71.0 |
| **Asian or Asian British** | 285,126 | 6.50 |
| **Black, Black British, Caribbean or African** | 116,570 | 2.66 |
| **Mixed or multiple ethnic groups** | 63,024 | 1.44 |
| **Other ethnic group** | 94,693 | 2.16 |
| **Unknown** | 712,286 | 16.3 |

*SD = Standard deviation



For peer review only - http://bmjopen.bmj.com/site/about/guidelines.xhtml

# 4. DISCUSSION.

## 4.1. Summary.

We created comprehensive codelists for identifying marginalised groups of interest including ethnicity, LD, SMI and transgender records in EHRs. Compared with the commonly used alternatives, our codelists improved data capture by 76.6% for LD, 68.9% for SMI and 36.9% for transgender records. These codelists have been made publicly available for others to use in their own research: https://osf.io/8skze/. They are readily available for use in UK research, but need to be adapted for international research, where different code systems are used. We provide code descriptions, alongside codes, to help facilitate this process.

## 4.2. Strengths and limitations.

This study has several strengths. First, codelists were identified through multiple sources, including CPRD publications and code list repositories, maximising the chances of identifying potentially relevant codes. Second, instead of accepting codes at face value, a Delphi review was implemented, with potentially irrelevant codes removed or segregated as uncertainty variables. The segregated codes will aid researchers for use in sensitivity analyses, if they find it relevant to their projects. Outlining this process aims to not only create comprehensive codelists for use in inequalities research, but also helps future researchers follow these reproducible methods in generating codelists and making them available for others to use.

This study also has a number of limitations. First, it only used codes from code lists arising from CPRD publications, or uploaded to code list repositories, and, despite several safety netting procedures, it is possible not all relevant codes were captured. This is exemplified by the fact that only 29% of relevant publications made their code lists publicly available, meaning there is potential for some codes to be missed. Second, while we compared the comprehensiveness of our codelists against commonly used alternatives (e.g. QOF), the comparisons were not like-for-like, due to differences in population definitions. For example, the QOF definition of SMI does not include severe major depression, and these differences may partially explain some of the increase observed in our codelist.

## 4.3. Comparison with previously published literature.

This research builds on the extant literature by synthesising the efforts of those who have previously curated code lists for the identification of marginalised groups. For example, Boyd et al [34] previously created a code list for identifying people who are transgender, which included a total of eight Read codes. Our list includes an additional 30 Read codes (along with the corresponding SNOMET-CT codes), enabling higher resolution and identification of people who are transgender.

## 4.4. Implications for research.

The findings of our study have several implications for research. Since only a small proportion of researchers made their codelists publicly available, there is a need to encourage researchers to share



For peer review only - http://bmjopen.bmj.com/site/about/guidelines.xhtml

their codelists. This will expedite inequalities research and prevent duplication of work. Second, while the percentages of ethnicity found in the population closely mirrored the frequencies reported in the UK census 2021 [18], we found that other marginalised groups were under-represented in the CPRD dataset (1.00%, 1.18% and 0.02% were recorded as having a LD, SMI, or transgender code, respectively, compared with national estimates of 2.16%, 2.00-3.00% and 0.20% respectively [35–37]). Further research is needed to understand why these frequencies do not match national estimates.

The implementation of our codelists in inequalities research will serve to simplify the complex process of identifying these groups of interest. The codelists will improve the extraction and management of diverse health information obtained from primary health care records, thus giving researchers a better ability to identify marginalised populations. This will boost the efficiency at which inequalities research is conducted using EHR.

## 5. CONCLUSION.

The outputs from our study provide an important contribution to future healthcare research since it is tackling the topic of addressing inequalities, which is held central to the NHS Long Term Plan for this decade [2]. With the combination of this work and previously published work developing codelists for marginalised populations [11–13], this will improve the representation of inequality groups in future research using EHR. We encourage others to use and edit our codelists as they see fit, and to follow our methods to develop comprehensive codelists for the identification of other groups that may be of interest to their research topics.

## REFERENCES.

1 What are healthcare inequalities? NHS Engl. 2021. https://www.england.nhs.uk/about/equality/equality-hub/national-healthcare-inequalities-improvement-programme/what-are-healthcare-inequalities/ (accessed 28 August 2024)

2 NHS Long Term Plan. NHS Engl. 2020. https://www.longtermplan.nhs.uk/ (accessed 4 September 2024)

3 Core20PLUS5 (adults) – an approach to reducing healthcare inequalities. NHS Engl. Digit. 2023. https://www.england.nhs.uk/about/equality/equality-hub/national-healthcare-inequalities-improvement-programme/core20plus5/ (accessed 28 August 2024)

4 Equality Act 2010. UK Gov. Legis. 2010. https://www.legislation.gov.uk/ukpga/2010/15/contents (accessed 4 September 2024)

5 Whitaker KL, Perchyk T, Kerrison RS, *et al.* Challenges in understanding inequities in help-seeking for possible cancer symptoms. *BMC Glob Public Health*. 2024;2:49. doi: 10.1186/s44263-024-00082-1

For peer review only



For peer review only - http://bmjopen.bmj.com/site/about/guidelines.xhtml

6 Electronic Health Records. Cent. Medicare Medicaid Serv. 2024. https://www.cms.gov/priorities/key-initiatives/e-health/records (accessed 4 September 2024)

7 Bhattacharyya S. *Introduction to SNOMED CT*. Singapore: Springer 2016.

8 Benchimol EI, Smeeth L, Guttmann A, *et al.* The REporting of studies Conducted using Observational Routinely-collected health Data (RECORD) statement. *PLoS Med*. 2015;12:e1001885. doi: 10.1371/journal.pmed.1001885

9 Matthewman J, Andresen K, Suffel A, *et al.* Checklist and guidance on creating codelists for electronic health records research. *NIHR Open Res*. 2024;4:20. doi: 10.3310/nihropenres.13550.1

10 Williams R, Kontopantelis E, Buchan I, *et al.* Clinical code set engineering for reusing EHR data for research: A review. *J Biomed Inform*. 2017;70:1–13. doi: 10.1016/j.jbi.2017.04.010

11 Hagberg KW, Jick SS. Validation of autism spectrum disorder diagnoses recorded in the Clinical Practice Research Datalink, 1990-2014. *Clin Epidemiol*. 2017;9:475–82. doi: 10.2147/CLEP.S139107

12 Kronk C, Tran GQ, Wu DTY. Creating a Queer Ontology: The Gender, Sex, and Sexual Orientation (GSSO) Ontology. *Stud Health Technol Inform*. 2019;264:208–12. doi: 10.3233/SHTI190213

13 Kuan V, Denaxas S, Patalay P, *et al.* Identifying and visualising multimorbidity and comorbidity patterns in patients in the English National Health Service: a population-based study. *Lancet Digit Health*. 2023;5:e16–27. doi: 10.1016/S2589-7500(22)00187-X

14 Watson J, Nicholson BD, Hamilton W, *et al.* Identifying clinical features in primary care electronic health record studies: methods for codelist development. *BMJ Open*. 2017;7:e019637. doi: 10.1136/bmjopen-2017-019637

15 NICE guidelines. Natl. Inst. Health Care Excell. 2024. https://www.nice.org.uk/about/what-we-do/our-programmes/nice-guidance/nice-guidelines (accessed 29 August 2024)

16 Ethnic group, national identity and religion. Off. Natl. Stat. 2021. https://www.ons.gov.uk/methodology/classificationsandstandards/measuringequality/ethnicgroupnationalidentityandreligion (accessed 4 September 2024)

17 Ethnicity. NHS Engl. Digit. 2022. https://digital.nhs.uk/data-and-information/data-collections-and-data-sets/data-sets/mental-health-services-data-set/submit-data/data-quality-of-protected-characteristics-and-other-vulnerable-groups/ethnicity (accessed 4 September 2024)

For peer review only

18 Ethnic group, England and Wales. Off. Natl. Stat. 2022. https://www.ons.gov.uk/peoplepopulationandcommunity/culturalidentity/ethnicity/bulletins/ethnicgroupenglandandwales/census2021 (accessed 30 August 2024)

19 Valuing People - A New Strategy for Learning Disability for the 21st Century. Dep. Health Soc. Care. 2001. https://www.gov.uk/government/publications/valuing-people-a-new-strategy-for-learning-disability-for-the-21st-century (accessed 4 September 2024)

20 Learning disability - applying All Our Health. Public Health Engl. 2023. https://www.gov.uk/government/publications/learning-disability-applying-all-our-health/learning-disabilities-applying-all-our-health (accessed 4 September 2024)

21 Karmiloff-Smith A, Al-Janabi T, D'Souza H, *et al.* The importance of understanding individual differences in Down syndrome. *F1000Research*. 2016;5:F1000 Faculty Rev-389. doi: 10.12688/f1000research.7506.1

22 Diagnostic and Statistical Manual of Mental Disorders (DSM-5-TR). Am. Psychiatr. Assoc. 2024. https://www.psychiatry.org:443/psychiatrists/practice/dsm (accessed 4 September 2024)

23 Trans people in the UK. Gov. Equal. Off. 2018. chrome-extension://efaidnbmnnnibpcajpcglclefindmkaj/https://assets.publishing.service.gov.uk/media/5b3a478240f0b64603fc181b/GEO-LGBT-factsheet.pdf (accessed 29 August 2024)

24 Bibliography. CPRD. 2024. https://www.cprd.com/bibliography (accessed 4 September 2024)

25 Clinical Practice Research Datalink. CPRD. 2024. https://www.cprd.com/ (accessed 4 September 2024)

26 OpenCodelists. OpenCodelists. 2022. https://www.opencodelists.org/ (accessed 28 August 2024)

27 HDRUK Phenotype Library. HDRUK Phenotype Libr. 2024. http://phenotypes.healthdatagateway.org/ (accessed 28 August 2024)

28 Department of Public Health and Primary Care. Univ. Camb. 2024. https://www.phpc.cam.ac.uk/pcu/research/researchgroups/crmh/cprd_cam/codelists/v11/ (accessed 28 August 2024)

29 Code list. LSHTM Data Compass. 2024. https://datacompass.lshtm.ac.uk/view/keywords/Code_list.html (accessed 4 September 2024)

30 Clinical coding – SNOMED CT. NHS Engl. 2023. https://www.england.nhs.uk/long-read/clinical-coding-snomed-ct/ (accessed 4 September 2024)

For peer review only

31 Hospital Episode Statistics (HES). NHS Engl. Digit. 2024. https://digital.nhs.uk/data-and-information/data-tools-and-services/data-services/hospital-episode-statistics (accessed 4 September 2024)

32 CPRD algorithm derived data. CPRD. 2024. https://www.cprd.com/cprd-algorithm-derived-data (accessed 30 August 2024)

33 Quality and Outcomes Framework (QOF). NHS Engl. Digit. 2023. https://digital.nhs.uk/data-and-information/data-tools-and-services/data-services/general-practice-data-hub/quality-outcomes-framework-qof (accessed 29 August 2024)

34 Boyd I, Hackett T, Bewley S. Care of Transgender Patients: A General Practice Quality Improvement Approach. *Healthc Basel Switz*. 2022;10:121. doi: 10.3390/healthcare10010121

35 How common is learning disability? Mencap. 2024. https://www.mencap.org.uk/learning-disability-explained/research-and-statistics/how-common-learning-disability (accessed 4 September 2024)

36 Mental health facts and statistics. Mind. 2020. https://www.mind.org.uk/information-support/types-of-mental-health-problems/mental-health-facts-and-statistics/ (accessed 4 September 2024)

37 Gender identity, England and Wales - Office for National Statistics: Census 2021. Off. Natl. Stat. 2023. https://www.ons.gov.uk/peoplepopulationandcommunity/culturalidentity/genderidentity/bulletins/genderidentityenglandandwales/census2021 (accessed 3 December 2024)

For peer review only

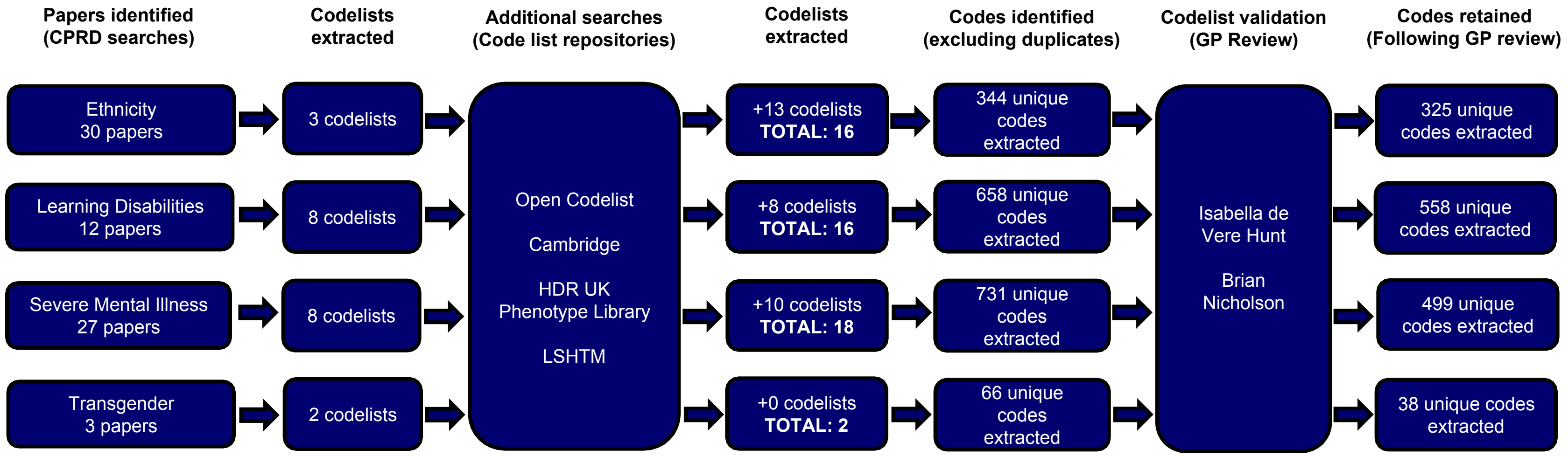
Papers identified (CPRD searches)
Codelists extracted
Additional searches (Code list repositories)
Codelists extracted
Codes identified (excluding duplicates)
Codelist validation (GP Review)
Codes retained (Following GP review)
Ethnicity 30 papers
3 codelists
+13 codelists TOTAL: 16
344 unique codes extracted
325 unique codes extracted
Learning Disabilities 12 papers
8 codelists
+8 codelists TOTAL: 16
658 unique codes extracted
558 unique codes extracted
Severe Mental Illness 27 papers
8 codelists
+10 codelists TOTAL: 18
731 unique codes extracted
499 unique codes extracted
Transgender 3 papers
2 codelists
+0 codelists TOTAL: 2
66 unique codes extracted
38 unique codes extracted
Open Codelist
Cambridge
HDR UK Phenotype Library
LSHTM
Isabella de Vere Hunt
Brian Nicholson

BMJ Open

# How well are marginalised groups represented in electronic records? A codelist development project and cross-sectional analysis of UK electronic health records.

| | |
|---|---|
| Journal: | *BMJ Open* |
| Manuscript ID | bmjopen-2024-098305.R1 |
| Article Type: | Original research |
| Date Submitted by the Author: | 04-Jun-2025 |
| Complete List of Authors: | Perchyk, Tetyana; University of Surrey, School of Health Sciences<br>de Vere Hunt, Isabella; University of Oxford, Nuffield Department of Primary Care Health Sciences<br>Nicholson, Brian; University of Oxford, Nuffield Department of Primary Care Health Sciences<br>Mounce, Luke; University of Exeter, Medical School<br>Sykes, Kate; Northumbria University, Faculty of Health and Lide Sciences<br>Lyratzopoulos, Georgios ; University College London, Department of Behavioural Science and Health<br>Lemanska, Agnieszka; University of Surrey; National Physical Laboratory, Data Science Department<br>Whitaker, Katriina; University of Surrey, School of Health Sciences<br>Kerrison, Robert; University of Surrey, School of Health Sciences |
| <b>Primary Subject Heading</b>: | General practice / Family practice |
| Secondary Subject Heading: | Epidemiology, Evidence based practice, Public health, Mental health |
| Keywords: | Primary Care < Primary Health Care, EPIDEMIOLOGY, MENTAL HEALTH, Transgender Persons |
| | |

SCHOLARONE™
Manuscripts

*I, the Submitting Author has the right to grant and does grant on behalf of all authors of the Work (as defined in the below author licence), an exclusive licence and/or a non-exclusive licence for contributions from authors who are: i) UK Crown employees; ii) where BMJ has agreed a CC-BY licence shall apply, and/or iii) in accordance with the terms applicable for US Federal Government officers or employees acting as part of their official duties; on a worldwide, perpetual, irrevocable, royalty-free basis to BMJ Publishing Group Ltd ("BMJ") its licensees and where the relevant Journal is co-owned by BMJ to the co-owners of the Journal, to publish the Work in this journal and any other BMJ products and to exploit all rights, as set out in our licence.*

*The Submitting Author accepts and understands that any supply made under these terms is made by BMJ to the Submitting Author unless you are acting as an employee on behalf of your employer or a postgraduate student of an affiliated institution which is paying any applicable article publishing charge ("APC") for Open Access articles. Where the Submitting Author wishes to make the Work available on an Open Access basis (and intends to pay the relevant APC), the terms of reuse of such Open Access shall be governed by a Creative Commons licence – details of these licences and which Creative Commons licence will apply to this Work are set out in our licence referred to above.*

*Other than as permitted in any relevant BMJ Author's Self Archiving Policies, I confirm this Work has not been accepted for publication elsewhere, is not being considered for publication elsewhere and does not duplicate material already published. I confirm all authors consent to publication of this Work and authorise the granting of this licence.*

For peer review only

**TITLE.** How well are marginalised groups represented in electronic records? A codelist development project and cross-sectional analysis of UK electronic health records.

**Corresponding author.** Tetyana Perchyk[1] (ORCID: 0009-0002-0273-8087)

**Co-authors.** Isabella de Vere Hunt[2] (ORCID: 0000-0002-4238-0924), Brian D Nicholson[2] (ORCID: 0000-0003-0661-7362), Luke Mounce[3] (ORCID: 0000-0002-6089-0661), Kate Sykes[4] (ORCID: 0000-0002-2942-7193), Georgios Lyratzopoulos[5] (ORCID: 0000-0002-2873-7421), Agnieszka Lemanska[1,6] (ORCID: 0000-0003-4849-2430)[1], Katriina L Whitaker[1] (ORCID: 0000-0002-0947-1840) and Robert S Kerrison[1] (ORCID: 0000-0002-8900-749X)

**Affiliations.** [1]University of Surrey, School of Health Sciences, United Kingdom; [2]University of Oxford, Nuffield Department of Primary Care Health Sciences, United Kingdom; [3]University of Exeter, Medical School, United Kingdom; [4]Northumbria University, Faculty of Health and Life Sciences, United Kingdom; [5]UCL, Department of Behavioural Science and Health, United Kingdom; [6]National Physical Laboratory, Data Science Department, United Kingdom.

**Contributorship Statement.** Tetyana Perchyk: Conceptualization, Methodology, Formal analysis, Investigation, Writing - Review & Editing, Guarantor.
Isabella de Vera Hunt: Fromal analysis, Writing - Reviewing & Editing.
Brian D Nicholson: Fromal analysis, Writing - Reviewing & Editing.
Luke Mounce: Formal analysis, Writing - Reviewing & Editing.
Kate Sykes: Conceptualization, Methodology, Writing - Reviewing & Editing.
Georgios Lyratzopoulos: Conceptualization, Methodology, Writing - Reviewing & Editing.
Agnieszka Lemanska: Conceptualization, Methodology, Writing - Reviewing & Editing.
Katriina Whitaker: Conceptualization, Writing - Reviewing & Editing.
Robert S Kerrison: Conceptualization, Methodology, Writing - Original draft, Supervision, Funding acquisition

**Funding.** The study was funded by Breast Cancer Now (reference: 2023FebIFS1615), the National Physical Laboratory (reference: NA) and the NIHR Policy Research Programme (reference: PR-PRU-NIHR206132. The views expressed are those of the author(s) and not necessarily those of the NIHR or the Department of Health and Social Care.

**Ethical approval.** This project did not involve the collection of primary data. As such, ethical approval was not required.

**Data availability.** The codelists developed through this work are available from Open Science Framework: https://osf.io/8skze/



For peer review only - http://bmjopen.bmj.com/site/about/guidelines.xhtml

**Competing interests.** The authors have no conflicts of interest to declare.



For peer review only - http://bmjopen.bmj.com/site/about/guidelines.xhtml

**ABSTRACT.**

**Objectives.** Primary care electronic health records provide a rich source of information for inequalities research. However, the reliability and validity of the research derived from these records depends on the completeness and resolution of the codelists (i.e. collection of medical terms/codes) used to identify populations of interest. The aim of this project was to develop comprehensive codelists for identifying people from ethnic minority groups, people with learning disabilities (LD), people with severe mental illness (SMI) and people who are transgender.

**Design.** We followed a three-stage process to define and extract relevant codelists. First, groups of interest were defined a priori. Next, relevant clinical codes, relating to the groups, were identified by searching Clinical Practice Research Datalink (CPRD) publications, codelist repositories and the CPRD Code Browser. Relevant codelists were extracted and merged according to group, and duplicates were removed. Finally, remaining codes were reviewed by two general practitioners.

**Setting.** The curated codelists were compared using a representative sample in the United Kingdom. The frequencies of individuals identified using the curated codelists were assessed and compared to widely used alternative codelists.

**Participants.** Comprehensiveness was assessed in a representative CPRD population of 10,966,759 people.

**Results.** After removal of duplicated and GP review, codelists were finalized with 325 unique codes for ethnicity, 558 for LD, 499 for SMI, and 38 for transgender. Compared with comparator codelists, an additional 48,017 (76.6%), 52,953 (68.9%) and 508 (36.9%) people with a LD, SMI or transgender code were identified. The proportions identified for ethnicity were consistent with expectations for the UK population with 71% white, 6.1% Asian, 2.7% black, 1.4% mixed, and 2.2% other.

**Conclusions.** The curated codelists are more sensitive than those widely used in practice and research. The codelists curated through this project will improve inequalities research by improving standards of identifying marginalised groups in primary care data.



For peer review only - http://bmjopen.bmj.com/site/about/guidelines.xhtml

**STRENGTHS AND LIMITATIONS.**

- This study followed standardised methodology for developing codelists, enhancing the scientific rigour, transparency and reproducibility of the study.
- Codelists were validated against existing codelists, using a representative sample of primary care medical records, comprising >10million patients.
- Only two general practitioners reviewed the codelists, neither of whom were experts in learning disabilities nor severe mental illness. As such, it is possible some conditions were overlooked or under-represented.
- Comparisons with existing codelists were not like-for-like due to differences in population definitions.

or peer review only

## MAIN TEXT

### 1. INTRODUCTION.

Health inequalities are unfair and avoidable differences in health, which are systematically established and arise from the social conditions in which people are born, grow, live, work and age [1].

In the United Kingdom (UK), health inequalities are a national priority [2]. The National Health Service for England (NHS England) developed 'The CORE20Plus5 Framework', which outlines priority groups and clinical areas requiring accelerated improvement [3]. The 'Core20' refers to the 20% most deprived areas in England, while the 'Plus' refers to population groups that should be identified at a local level, including (but not limited to): ethnic minority groups, people with a learning disability (LD) or autism, people with multiple long-term health conditions, other groups that share protected characteristics (as defined by the Equality Act 2010 [4]), and groups experiencing social exclusion (such as coastal communities, where there may be small areas of high deprivation hidden amongst relative affluence) [3]. The '5' describes key clinical areas requiring accelerated improvement for these populations, namely: maternity, severe mental illness (SMI), chronic respiratory disease, early cancer diagnosis and hypertension case-finding and management [3].

While the CORE20Plus5 framework provides a useful outline of national priorities for health research and clinical practice, there are several ongoing challenges that need to be addressed for researchers to support national strategy [5]. Pertinent among these is the identification and classification of CORE20Plus groups (also referred to as 'socially disadvantaged' or 'marginalised' groups) within electronic health records (EHRs) [5]. EHRs include key administrative clinical data relevant to a person's care, including demographics, progress notes, problems, medications, vital signs, past medical history, immunisations, laboratory data and radiology reports [6]. These data are recorded using clinical coding systems, typically containing hundreds of thousands of concepts [7], which need to be categorised via codelists (i.e. collection of medical terms/codes) to facilitate research.

While methods for codelist development and reporting have previously been published [8–10], and many studies now report the methods used to develop codelists for various medical diagnoses, few papers describe the systematic development of codelists specifically for identifying marginalised populations [9]. Some papers have attempted to develop codelists for certain marginalised groups, including for identifying the autistic population [11], the LGB population [12], and for people who experience multiple long-term conditions [13]. However, there does not appear to be any papers describing the development of codelists for other marginalised groups.

To support future health inequalities research, we set out to develop comprehensive codelists for the identification of four CORE20plus5 groups, namely: people from ethnic minority groups, people with LDs (excluding autistic people), people with SMI and people who are transgender. We selected these



For peer review only - http://bmjopen.bmj.com/site/about/guidelines.xhtml

groups because there is a general lack of standardised methods for codelist development to describe these inequality populations in EHRs.

## 2. METHOD.

Previously published approaches for codelist development were reviewed [9,10], and Watson et al's [14] approach was deemed the most appropriate because it specifically sets out methods for developing codelists using UK based EHRs.

The approach comprises three steps: **Step 1.** Clearly define the clinical feature of interest a priori; **Step 2.** Assemble a list of codes that may be used to record the clinical feature; **Step 3.** Expert review of codes.

The following presents a detailed overview of the processes undertaken for each step.

### 2.1. **Step 1.** Clearly define the clinical feature of interest a priori.

Watson et al recommend beginning by clearly defining the clinical feature of interest [14]. To do this, they recommend using reliable sources of information, such as the National Institute for Health and Care Excellence (NICE) [15]. For the purposes of this study, we used the following definitions and sources to define marginalised groups of interest:

**Ethnicity.** The Office of National Statistics (ONS) [16] has highlighted that there is no true consensus on what defines an ethnic group, as identification to these are "self-defined and subjectively meaningful to the individual". However, as stated by NHS England, it is generally accepted that ethnicity includes a variety of elements, such as ancestry, culture, identity, religion, language, and physical appearance [17].

We defined ethnicity according to the characteristics set out in the Equality Act 2010, namely: colour, nationality and ethnic or national origins [4]. To this end, UK Census 2021 categories were used to group ethnicity codes (see Table 1) [18].



For peer review only - http://bmjopen.bmj.com/site/about/guidelines.xhtml

**Table 1.** UK census ethnic categories [15].

| |
|---|
| **Asian or Asian British** |
| Indian |
| Pakistani |
| Bangladeshi |
| Chinese |
| Any other Asian background |
| **Black, Black British, Caribbean or African** |
| Caribbean |
| African |
| Any other Black, Black British, or Caribbean background |
| **Mixed or multiple ethnic groups** |
| White and Black Caribbean |
| White and Black African |
| White and Asian |
| Any other Mixed or multiple ethnic background |
| **White** |
| English, Welsh, Scottish, Northern Irish or British |
| Irish |
| Gypsy or Irish Traveller |
| Roma |
| Any other White background |
| **Other ethnic group** |
| Arab |
| Any other ethnic group |



For peer review only - http://bmjopen.bmj.com/site/about/guidelines.xhtml

**Learning disability.** The Department of Health and Social Care (DHSC) [19] defines a learning disability as: “a significantly reduced ability to understand new or complex information, to learn new skills (impaired intelligence), with a reduced ability to cope independently (impaired social functioning), which started before adulthood.” According to Public Health England [20]: “a learning disability is different to a learning difficulty, which is a reduced intellectual ability for a specific form of learning and includes conditions such as dyslexia (reading), dyspraxia (affecting physical co-ordination) and attention deficit hyperactivity disorder (ADHD).”

We defined learning disabilities according to the characteristics described by the DHSC. This is because there is significant overlap between a diagnosis of learning disability and learning difficulty, and these two terms have historically been used interchangeably. To capture the most accurate population experiencing inequalities and to avoid possible misclassifications, the more inclusive DHSC definition was used to describe the LD population. This definition also excludes autism, which is a separate diagnosis (a higher proportion of people with a learning disability have autism, compared with the general population, but not everyone with autism has a learning disability). We did not categorise learning disabilities into groups. They are often described as being ‘mild’, ‘moderate’, or ‘severe’, ‘verbal’ or ‘non-verbal’, and ‘syndromic’ or ‘non-syndromic’, but there is no consensus as to which learning disabilities fall into which categories, and there is individual-level variation within the same disability, making it inappropriate to generalise [21].

**Severe mental illness.** The Diagnostic and Statistical Manual, fifth edition (DSM-V), defines severe mental illness as: “a mental, behavioural, or emotional disorder resulting in serious functional impairment, which substantially interferes with or limits one or more major life activities.” [22].

We defined SMI according to the characteristics described in the DSM-V, and categorised codes according to four recognised conditions, namely: ‘schizophrenia’, ‘bipolar disorder’, ‘severe major depression’ and ‘other psychotic disorders’ [22].

**Transgender.** The UK Government Equality Office [23] defines trans (transgender) people as “People whose gender is different from the gender assigned to them at birth.” They provide the following by way of example: “A trans man is someone that transitioned from woman to man” [23].

We used the definition provided by the Government Equality Office [23]. Most clinical codes do not differentiate between trans men and trans women; therefore, we did not group codes into these categories.

**Patient and public involvement.**

Five members of the public with lived experience of autism, severe mental illness, minoritised ethnicity and/or being transgender contributed to the study. While no one with a learning disability was included,

a person with autism was able to provide perspective on the differences between autism and learning disability, and their lived experience of the two being conflated with one another.

Initial plans and findings were shared with these collaborators during quarterly meetings. During these meetings, collaborators provided feedback on the codelists, the language used to define them, and how to handle outdated or offensive terminology (such terms were retained within the codelist to ensure completeness but were omitted from the manuscript, so as not to perpetuate their use in medical literature.

**2.2. Step 2.** Assemble list of codes that may be used to record the clinical feature.

In the second step, an online thesaurus was used to develop a list of synonyms associated with the clinical features of interest. An overview of the synonyms used for each clinical feature is presented in Table 2.

**Table 2.** List of synonyms used for each clinical feature

| Clinical feature | Synonyms |
|---|---|
| **Ethnicity** | Nationality, Nationalities, Nation(s), Race(s), Racial, Colour, Ethnic, Ethnicity, Minority, Minorities |
| **Learning Disability** | Intellectual disability, Intellectual disabilities, Learning disability, Learning Disabilities, ID(s), LD(s) |
| **Severe mental illness** | Serious mental illness, Serious mental illnesses, Severe mental illness, Severe mental illnesses, SMI(s), Schiz*, Psychosis, Bipolar, Major depress* |
| **Transgender** | Trans, Transsexual, Transgender, Transgender, Gender dysmorphia, Trans man, Trans woman, Trans men, Trans women, Transfeminine, Transmasculine, Gender Surgery, LGBT. |

These identified synonyms were used to search literature and databases to find relevant published codelists related to the inequality groups. One major resource used was the October 2023 Clinical Practice Research Datalink (CPRD) Bibliography [24]. CPRD is one of the largest research databases of electronic, anonymised, and longitudinal medical records from primary care in the world [25]. The CPRD Bibliography is a resource which provides a record of all published studies (>3,000 as of October 2023) using CPRD data, and this aided in our search for publications. The relevant publications were identified using our list of synonyms and codelists used in these publications were subsequently downloaded, where they had been made available.

To capture codelists used in non-CPRD studies, we also searched for, and downloaded, relevant codelists from four widely used codelist repositories, namely: OpenCodelists [26], the Health Data



For peer review only - http://bmjopen.bmj.com/site/about/guidelines.xhtml

Research UK (HDRUK) Phenotype Library [27], the University of Cambridge Codelist Repository [28] and the London School of Hygiene and Tropical Medicine (LSHTM) Data Compass [29].

Once all relevant published codelists were identified, they were combined into one excel file for each inequality group. This gave us four files: one for ethnicity, LD, SMI, and transgender. Within each of these filed, any duplicate codes were removed, and remaining codes grouped by the two main coding systems used in the UK: Read and systematized nomenclature of medicine clinical terminology (SNOMED-CT). Read was generally used in primary care until 2018, and SNOMED-CT thereafter, following the enablement of SNOMED-CT within Primary care systems [30]. This provided an aggregated list encompassing every code (excluding duplicates) that has been used and published wither through CPRD literature or published through an online codelist database.

These aggregated codelists were then checked for accuracy using the CPRD Code Browser, which is a tool specific to CPRD and is provided to researchers who gain access to their data. This tool contains the diagnostic description, the alphanumeric Read / SNOMED-CT code originally used by the general practitioner (GP) to enter the clinical information, and CPRD's proprietary 'medcode' / 'medcodeid' (which is simply a numeric equivalent of the Read / SNOMED-CT code) [30]. The tool allowed for easy browsing by either using a known code or description to check code accuracy. Searching by the medical descriptors for the code, or "terms," was useful because each search provided a list of all possible codes. For example, a search for "psychosis" showed every diagnostic term and associated code available from primary care GP records. This was useful to cross reference each of the individual codes and ensure all relevant options have been included. In line with the method described by Watson et al [14], inaccurate codes were removed or corrected, and additional codes (not previously identified) added if they were associated with the diagnostic definition of one or more eligible codes.

### 2.3. **Step 3.** Expert review of codes.

In the final step, the codelists were sent to a GP (IVH) for review. They scored each code using the following three-point scale:

1. Definitely include: the code accurately defines the clinical feature of interest, and GPs would use it.
2. Uncertain: it remains unclear whether the code accurately reflects the clinical feature of interest, or whether GPs would use it.
3. Definitely exclude: the code does not define the clinical feature of interest, and GPs would avoid.

Codes assigned a score of 2 ('Uncertain') or 3 ('Definitely exclude') were reviewed by a second GP (BN). All codes assigned a score of 3 ('Definitely exclude') by both reviewers were removed from the codelists. All codes receiving a score of 2 from either reviewer were retained as 'uncertainty variables',

which are recommended for sensitivity analyses [14]. These variables are typically less frequently used by GPs, but may identify additional, potentially relevant, cases [14].

### 2.4. Assessing codelist comprehensiveness

To explore the comprehensiveness of the developed codelists, we used the CPRD database to ascertain the number of people with at least one recording of an included code for each list. We restricted the population to those who had been registered with their CPRD Aurum practice since at least 1st January 2019 and who had neither died nor deregistered from their practice by August 2022 (the date of last data collection).

In the context of CPRD studies, researchers have been able to acquire already categorised ethnicity data through linkage with the Hospital Episode Statistic (HES) [31]. Derived ethnicity data is also now available through CPRD at an additional cost [32]. The ethnicity codelist we generated will service to those researchers who are using other non-CPRD datasets, or ones who may not have access to either the linked HES data or the CPRD derived ethnicity.

For the other variables, validated codelists from other sources were selected as comparisons, seeking to match the scope of each list as closely as possible. Codelists for LD and SMI were sourced from the Quality and Outcomes Framework (QOF: the pay for performance scheme for general practices in the UK) [33]. QOF includes incentives for practices to keep a register of patients with certain conditions, including LD and SMI, and produces validated codelists for this purpose. We used lists from the QOF Business Rules version 45; “Learning Disability codes” (list name LD_COD) and "Psychosis and schizophrenia and bipolar affective disease codes" (list MH_COD) as our comparison lists. For the transgender category, no QOF list exists, and literature is slim. We identified two identical previously published codelists that were used to compare to our newly developed codelist.

## 3. RESULTS.

A review of 87 relevant publications and additional codelist repositories identified a total of 55 codelists: 16 for ethnicity, 16 for LD, 21 for SMI and 2 for transgender. After removing duplicates and expert review, 388 codes were excluded, and 1,420 total unique codes remained: 325 codes for ethnicity, 558 codes for LD, 499 codes for SMI and 38 codes for transgender. The full review process is demonstrated in figure 1.

(Figure 1 Here)

The codes were run in the source population of CPRD (n=10,966,759) to ascertain the number of people captured by the codelists (see Table 3). People were identified by the presence of one or more codes related to the inequality group of interest. Ethnicity data were available for 4,384,017 (40.0%) individuals. Of these, 3,112,319 (71.0%) were ethnically white; 285,126 (6.50%) were ethnically Asian;



For peer review only - http://bmjopen.bmj.com/site/about/guidelines.xhtml

116,570 (2.66%) were ethnically black; 63,024 (1.44%) were of mixed ethnicity; 94,693 (2.16%) were categorised as ‘other;’ and 712,286 (16.3%) were of unknown ethnicity.

110,692 people were found to have LD (1.00%), 129,788 people had SMI (1.18%) and 1,884 people were transgender (0.02%). This represented an increased capture of 48,017 (76.6%) for LD, 52,953 (68.9%) for SMI, and 508 (36.9%) for transgender (see Table 4).

For peer review only



For peer review only - http://bmjopen.bmj.com/site/about/guidelines.xhtml

**Table 3.** Descriptives for CPRD population, including frequencies between ethnic groups derived from our curated codelist.

| Population Total (N) | Mean age (SD*) | Age range | Frequency males (%) | Frequency females (%) |
|---|---|---|---|---|
| 10,966,759 | 43.3 (22.8) | 4 – 100 years old | 5,554,024 (50.6) | 5,412,735 (49.4) |
| **Ethnicity** | **Frequency from curated codelist (N)** | | **Frequency percent (%)** | |
| **White** | 3,112,319 | | 71.0 | |
| **Asian or Asian British** | 285,126 | | 6.50 | |
| **Black, Black British, Caribbean or African** | 116,570 | | 2.66 | |
| **Mixed or multiple ethnic groups** | 63,024 | | 1.44 | |
| **Other ethnic group** | 94,693 | | 2.16 | |
| **Unknown** | 712,286 | | 16.3 | |

*SD = Standard deviation

For peer review only

**Table 4.** Frequencies of populations extracted from CPRD, comparing original and curated codelist for LD, SMI and transgender groups.

| Inequality Group | Original Codelist (N) | Curated Codelist (N) | Difference in Identification (N) | Percent Increase (%) |
|---|---|---|---|---|
| **Learning disability** | 62,675 | 110,692 | 48,017 | 76.6 |
| **Severe mental illness** | 76,835 | 129,788 | 52,953 | 68.9 |
| **Transgender** | 1,376 | 1,884 | 508 | 36.9 |

## 4. DISCUSSION.

### 4.1. Summary.

We created comprehensive codelists for identifying marginalised groups of interest including ethnicity, LD, SMI and transgender records in EHRs. Compared with the commonly used alternatives, our codelists improved data capture by 76.6% for LD, 68.9% for SMI and 36.9% for transgender records. These codelists have been made publicly available for others to use in their own research: https://osf.io/8skze/ [34]. They are readily available for use in UK research, but need to be adapted for international research, where different code systems are used. We provide code descriptions, alongside codes, to help facilitate this process.

### 4.2. Strengths and limitations.

This study has several strengths. First, codelists were identified through multiple sources, including CPRD publications and code list repositories, maximising the chances of identifying potentially relevant codes. Second, instead of accepting codes at face value, an expert review was implemented, with potentially irrelevant codes removed or segregated as uncertainty variables. The segregated codes will aid researchers for use in sensitivity analyses, if they find it relevant to their projects.

This study also has a number of limitations. First, it only used codes from code lists arising from CPRD publications, or uploaded to code list repositories, and, despite several safety netting procedures, it is possible not all relevant codes were captured. This is exemplified by the fact that only 29% of relevant publications made their code lists publicly available, meaning there is potential for some codes to be missed. Second, while we compared the comprehensiveness of our codelists against commonly used alternatives (e.g. QOF), the comparisons were not like-for-like, due to differences in population definitions. For example, the QOF definition of SMI does not include severe major depression, and these differences may partially explain some of the increase observed in our codelist. Finally, only two experts reviewed the codelists, both of whom were general practitioners. While a strength in terms of them being representative of the population responsible for entering medical codes in primary care clinical systems, they may be less familiar with the plethora of medical diagnoses associated with some conditions such as SMI and LD, which may have resulted in some conditions being omitted from our codelists. We encourage experts (e.g. learning disability nurse) to feedback and refine these lists.

### 4.3. Comparison with previously published literature.

This research builds on the extant literature by synthesising the efforts of those who have previously curated code lists for the identification of marginalised groups. For example, Boyd et al [35] previously created a code list for identifying people who are transgender, which included a total of eight Read codes. Our list includes an additional 30 Read codes (along with the corresponding SNOMET-CT codes), enabling higher resolution and identification of people who are transgender.

### 4.4. Implications for research.

The findings of our study have several implications for research. Since only a small proportion of researchers made their codelists publicly available, there is a need to encourage researchers to share their codelists. This will expedite inequalities research and prevent duplication of work. Second, while the percentages of ethnicity found in the population closely mirrored the frequencies reported in the UK census 2021 [18], we found that other marginalised groups were under-represented in the CPRD dataset (1.00%, 1.18% and 0.02% were recorded as having a LD, SMI, or transgender code, respectively, compared with national estimates of 2.16%, 2.00-3.00% and 0.20% respectively [36–38]). Further research is needed to understand why these frequencies do not match national estimates.

The implementation of our codelists in inequalities research will serve to simplify the complex process of identifying these groups of interest. The codelists will improve the extraction and management of diverse health information obtained from primary health care records, thus providing researchers with flexible tools that can be adapted to identify marginalised populations in accordance with local definitions. This will boost the efficiency at which inequalities research is conducted using EHR.

## 5. CONCLUSION.

The outputs from our study provide an important contribution to future healthcare research since it is tackling the topic of addressing inequalities, which is held central to the NHS Long Term Plan for this decade [2]. With the combination of this work and previously published work developing codelists for marginalised populations [11–13], this will improve the representation of inequality groups in future research using EHR. We encourage others to use and edit our codelists as they see fit, and to follow our methods to develop comprehensive codelists for the identification of other groups that may be of interest to their research topics.

## REFERENCES.

1 What are healthcare inequalities? NHS Engl. 2021. https://www.england.nhs.uk/about/equality/equality-hub/national-healthcare-inequalities-improvement-programme/what-are-healthcare-inequalities/ (accessed 28 August 2024)

2 NHS Long Term Plan. NHS Engl. 2020. https://www.longtermplan.nhs.uk/ (accessed 4 September 2024)

3 Core20PLUS5 (adults) – an approach to reducing healthcare inequalities. NHS Engl. Digit. 2023. https://www.england.nhs.uk/about/equality/equality-hub/national-healthcare-inequalities-improvement-programme/core20plus5/ (accessed 28 August 2024)

4 Equality Act 2010. UK Gov. Legis. 2010. https://www.legislation.gov.uk/ukpga/2010/15/contents (accessed 4 September 2024)

5 Whitaker KL, Perchyk T, Kerrison RS, *et al.* Challenges in understanding inequities in help-seeking for possible cancer symptoms. *BMC Glob Public Health*. 2024;2:49. doi: 10.1186/s44263-024-00082-1

6 Electronic Health Records. Cent. Medicare Medicaid Serv. 2024. https://www.cms.gov/priorities/key-initiatives/e-health/records (accessed 4 September 2024)



For peer review only - http://bmjopen.bmj.com/site/about/guidelines.xhtml

7 Bhattacharyya S. *Introduction to SNOMED CT*. Singapore: Springer 2016.

8 Benchimol EI, Smeeth L, Guttmann A, *et al.* The REporting of studies Conducted using Observational Routinely-collected health Data (RECORD) statement. *PLoS Med*. 2015;12:e1001885. doi: 10.1371/journal.pmed.1001885

9 Matthewman J, Andresen K, Suffel A, *et al.* Checklist and guidance on creating codelists for electronic health records research. *NIHR Open Res*. 2024;4:20. doi: 10.3310/nihropenres.13550.1

10 Williams R, Kontopantelis E, Buchan I, *et al.* Clinical code set engineering for reusing EHR data for research: A review. *J Biomed Inform*. 2017;70:1–13. doi: 10.1016/j.jbi.2017.04.010

11 Hagberg KW, Jick SS. Validation of autism spectrum disorder diagnoses recorded in the Clinical Practice Research Datalink, 1990-2014. *Clin Epidemiol*. 2017;9:475–82. doi: 10.2147/CLEP.S139107

12 Kronk C, Tran GQ, Wu DTY. Creating a Queer Ontology: The Gender, Sex, and Sexual Orientation (GSSO) Ontology. *Stud Health Technol Inform*. 2019;264:208–12. doi: 10.3233/SHTI190213

13 Kuan V, Denaxas S, Patalay P, *et al.* Identifying and visualising multimorbidity and comorbidity patterns in patients in the English National Health Service: a population-based study. *Lancet Digit Health*. 2023;5:e16–27. doi: 10.1016/S2589-7500(22)00187-X

14 Watson J, Nicholson BD, Hamilton W, *et al.* Identifying clinical features in primary care electronic health record studies: methods for codelist development. *BMJ Open*. 2017;7:e019637. doi: 10.1136/bmjopen-2017-019637

15 NICE guidelines. Natl. Inst. Health Care Excell. 2024. https://www.nice.org.uk/about/what-we-do/our-programmes/nice-guidance/nice-guidelines (accessed 29 August 2024)

16 Ethnic group, national identity and religion. Off. Natl. Stat. 2021. https://www.ons.gov.uk/methodology/classificationsandstandards/measuringequality/ethnicgroupnationalidentityandreligion (accessed 4 September 2024)

17 Ethnicity. NHS Engl. Digit. 2022. https://digital.nhs.uk/data-and-information/data-collections-and-data-sets/data-sets/mental-health-services-data-set/submit-data/data-quality-of-protected-characteristics-and-other-vulnerable-groups/ethnicity (accessed 4 September 2024)

18 Ethnic group, England and Wales. Off. Natl. Stat. 2022. https://www.ons.gov.uk/peoplepopulationandcommunity/culturalidentity/ethnicity/bulletins/ethnicgroupenglandandwales/census2021 (accessed 30 August 2024)

19 Valuing People - A New Strategy for Learning Disability for the 21st Century. Dep. Health Soc. Care. 2001. https://www.gov.uk/government/publications/valuing-people-a-new-strategy-for-learning-disability-for-the-21st-century (accessed 4 September 2024)

20 Learning disability - applying All Our Health. Public Health Engl. 2023. https://www.gov.uk/government/publications/learning-disability-applying-all-our-health/learning-disabilities-applying-all-our-health (accessed 4 September 2024)

21 Karmiloff-Smith A, Al-Janabi T, D'Souza H, *et al.* The importance of understanding individual differences in Down syndrome. *F1000Research*. 2016;5:F1000 Faculty Rev-389. doi: 10.12688/f1000research.7506.1

22 Diagnostic and Statistical Manual of Mental Disorders (DSM-5-TR). Am. Psychiatr. Assoc. 2024. https://www.psychiatry.org:443/psychiatrists/practice/dsm (accessed 4 September 2024)

For peer review only



For peer review only - http://bmjopen.bmj.com/site/about/guidelines.xhtml

23 Trans people in the UK. Gov. Equal. Off. 2018. chrome-extension://efaidnbmnnnibpcajpcglclefindmkaj/https://assets.publishing.service.gov.uk/media/5b3a478240f0b64603fc181b/GEO-LGBT-factsheet.pdf (accessed 29 August 2024)

24 Bibliography. CPRD. 2024. https://www.cprd.com/bibliography (accessed 4 September 2024)

25 Clinical Practice Research Datalink. CPRD. 2024. https://www.cprd.com/ (accessed 4 September 2024)

26 OpenCodelists. OpenCodelists. 2022. https://www.opencodelists.org/ (accessed 28 August 2024)

27 HDRUK Phenotype Library. HDRUK Phenotype Libr. 2024. http://phenotypes.healthdatagateway.org/ (accessed 28 August 2024)

28 Department of Public Health and Primary Care. Univ. Camb. 2024. https://www.phpc.cam.ac.uk/pcu/research/researchgroups/crmh/cprd_cam/codelists/v11/ (accessed 28 August 2024)

29 Code list. LSHTM Data Compass. 2024. https://datacompass.lshtm.ac.uk/view/keywords/Code_list.html (accessed 4 September 2024)

30 Clinical coding – SNOMED CT. NHS Engl. 2023. https://www.england.nhs.uk/long-read/clinical-coding-snomed-ct/ (accessed 4 September 2024)

31 Hospital Episode Statistics (HES). NHS Engl. Digit. 2024. https://digital.nhs.uk/data-and-information/data-tools-and-services/data-services/hospital-episode-statistics (accessed 4 September 2024)

32 CPRD algorithm derived data. CPRD. 2024. https://www.cprd.com/cprd-algorithm-derived-data (accessed 30 August 2024)

33 Quality and Outcomes Framework (QOF). NHS Engl. Digit. 2023. https://digital.nhs.uk/data-and-information/data-tools-and-services/data-services/general-practice-data-hub/quality-outcomes-framework-qof (accessed 29 August 2024)

[dataset] 34 Kerrison, RS. Code lists for inequalities research. Open Sci. Framew. 2024. https://doi.org/10.17605/OSF.IO/8SKZE (accessed 6 April 2025)

35 Boyd I, Hackett T, Bewley S. Care of Transgender Patients: A General Practice Quality Improvement Approach. *Healthc Basel Switz*. 2022;10:121. doi: 10.3390/healthcare10010121

36 How common is learning disability? Mencap. 2024. https://www.mencap.org.uk/learning-disability-explained/research-and-statistics/how-common-learning-disability (accessed 4 September 2024)

37 Mental health facts and statistics. Mind. 2020. https://www.mind.org.uk/information-support/types-of-mental-health-problems/mental-health-facts-and-statistics/ (accessed 4 September 2024)

38 Gender identity, England and Wales - Office for National Statistics: Census 2021. Off. Natl. Stat. 2023. https://www.ons.gov.uk/peoplepopulationandcommunity/culturalidentity/genderidentity/bulletins/genderidentityenglandandwales/census2021 (accessed 3 December 2024)

For peer review only

**Figure 1.** Process outlining codelist review and development.

For peer review only



For peer review only - http://bmjopen.bmj.com/site/about/guidelines.xhtml

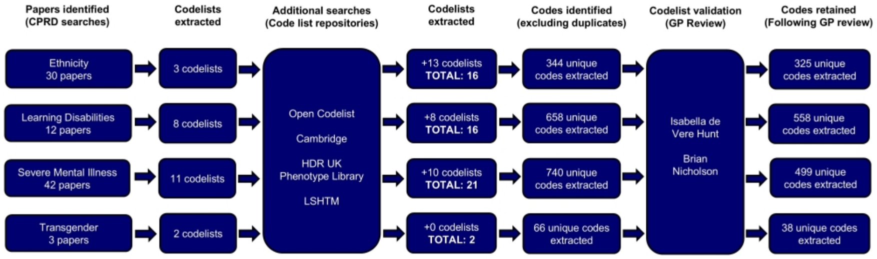


Caption : Figure 1. Process outlining codelist review and development.

338x190mm (300 x 300 DPI)

BMJ Open

# How well are marginalised groups represented in electronic records? A codelist development project and cross-sectional analysis of UK electronic health records.

| | |
|---|---|
| Journal: | *BMJ Open* |
| Manuscript ID | bmjopen-2024-098305.R2 |
| Article Type: | Original research |
| Date Submitted by the Author: | 26-Jun-2025 |
| Complete List of Authors: | Perchyk, Tetyana; University of Surrey, School of Health Sciences<br>de Vere Hunt, Isabella; University of Oxford, Nuffield Department of Primary Care Health Sciences<br>Nicholson, Brian; University of Oxford, Nuffield Department of Primary Care Health Sciences<br>Mounce, Luke; University of Exeter, Medical School<br>Sykes, Kate; Northumbria University, Faculty of Health and Lide Sciences<br>Lyratzopoulos, Georgios ; University College London, Department of Behavioural Science and Health<br>Lemanska, Agnieszka; University of Surrey; National Physical Laboratory, Data Science Department<br>Whitaker, Katriina; University of Surrey, School of Health Sciences<br>Kerrison, Robert; University of Surrey, School of Health Sciences |
| <b>Primary Subject Heading</b>: | General practice / Family practice |
| Secondary Subject Heading: | Epidemiology, Evidence based practice, Public health, Mental health |
| Keywords: | Primary Care < Primary Health Care, EPIDEMIOLOGY, MENTAL HEALTH, Transgender Persons |
| | |

SCHOLARONE™
Manuscripts

*I, the Submitting Author has the right to grant and does grant on behalf of all authors of the Work (as defined in the below author licence), an exclusive licence and/or a non-exclusive licence for contributions from authors who are: i) UK Crown employees; ii) where BMJ has agreed a CC-BY licence shall apply, and/or iii) in accordance with the terms applicable for US Federal Government officers or employees acting as part of their official duties; on a worldwide, perpetual, irrevocable, royalty-free basis to BMJ Publishing Group Ltd ("BMJ") its licensees and where the relevant Journal is co-owned by BMJ to the co-owners of the Journal, to publish the Work in this journal and any other BMJ products and to exploit all rights, as set out in our licence.*

*The Submitting Author accepts and understands that any supply made under these terms is made by BMJ to the Submitting Author unless you are acting as an employee on behalf of your employer or a postgraduate student of an affiliated institution which is paying any applicable article publishing charge ("APC") for Open Access articles. Where the Submitting Author wishes to make the Work available on an Open Access basis (and intends to pay the relevant APC), the terms of reuse of such Open Access shall be governed by a Creative Commons licence – details of these licences and which Creative Commons licence will apply to this Work are set out in our licence referred to above.*

*Other than as permitted in any relevant BMJ Author's Self Archiving Policies, I confirm this Work has not been accepted for publication elsewhere, is not being considered for publication elsewhere and does not duplicate material already published. I confirm all authors consent to publication of this Work and authorise the granting of this licence.*

For peer review only

**TITLE.** How well are marginalised groups represented in electronic records? A codelist development project and cross-sectional analysis of UK electronic health records.

**Corresponding author.** Tetyana Perchyk[1] (ORCID: 0009-0002-0273-8087)

**Co-authors.** Isabella de Vere Hunt[2] (ORCID: 0000-0002-4238-0924), Brian D Nicholson[2] (ORCID: 0000-0003-0661-7362), Luke Mounce[3] (ORCID: 0000-0002-6089-0661), Kate Sykes[4] (ORCID: 0000-0002-2942-7193), Georgios Lyratzopoulos[5] (ORCID: 0000-0002-2873-7421), Agnieszka Lemanska[1,6] (ORCID: 0000-0003-4849-2430)[1], Katriina L Whitaker[1] (ORCID: 0000-0002-0947-1840) and Robert S Kerrison[1] (ORCID: 0000-0002-8900-749X)

**Affiliations.** [1]University of Surrey, School of Health Sciences, United Kingdom; [2]University of Oxford, Nuffield Department of Primary Care Health Sciences, United Kingdom; [3]University of Exeter, Medical School, United Kingdom; [4]Northumbria University, Faculty of Health and Life Sciences, United Kingdom; [5]UCL, Department of Behavioural Science and Health, United Kingdom; [6]National Physical Laboratory, Data Science Department, United Kingdom.

**Contributorship Statement.** Tetyana Perchyk: Conceptualization, Methodology, Formal analysis, Investigation, Writing - Review & Editing, Guarantor.
Isabella de Vera Hunt: Fromal analysis, Writing - Reviewing & Editing.
Brian D Nicholson: Fromal analysis, Writing - Reviewing & Editing.
Luke Mounce: Formal analysis, Writing - Reviewing & Editing.
Kate Sykes: Conceptualization, Methodology, Writing - Reviewing & Editing.
Georgios Lyratzopoulos: Conceptualization, Methodology, Writing - Reviewing & Editing.
Agnieszka Lemanska: Conceptualization, Methodology, Writing - Reviewing & Editing.
Katriina Whitaker: Conceptualization, Writing - Reviewing & Editing.
Robert S Kerrison: Conceptualization, Methodology, Writing - Original draft, Supervision, Funding acquisition

**Funding.** The study was funded by Breast Cancer Now (reference: 2023FebIFS1615), the National Physical Laboratory (reference: NA) and the NIHR Policy Research Programme (reference: PR-PRU-NIHR206132. The views expressed are those of the author(s) and not necessarily those of the NIHR or the Department of Health and Social Care.

**Ethical approval.** This project did not involve the collection of primary data. As such, ethical approval was not required.

**Data availability.** The codelists developed through this work are available from Open Science Framework: https://osf.io/8skze/

**Competing interests.** The authors have no conflicts of interest to declare.



For peer review only - http://bmjopen.bmj.com/site/about/guidelines.xhtml

**ABSTRACT.**

**Objectives.** Primary care electronic health records provide a rich source of information for inequalities research. However, the reliability and validity of the research derived from these records depends on the completeness and resolution of the codelists (i.e. collection of medical terms/codes) used to identify populations of interest. The aim of this project was to develop comprehensive codelists for identifying people from ethnic minority groups, people with learning disabilities (LD), people with severe mental illness (SMI) and people who are transgender.

**Design.** We followed a three-stage process to define and extract relevant codelists. First, groups of interest were defined a priori. Next, relevant clinical codes, relating to the groups, were identified by searching Clinical Practice Research Datalink (CPRD) publications, codelist repositories and the CPRD Code Browser. Relevant codelists were extracted and merged according to group, and duplicates were removed. Finally, remaining codes were reviewed by two general practitioners.

**Setting.** The curated codelists were compared using a representative sample in the United Kingdom. The frequencies of individuals identified using the curated codelists were assessed and compared to widely used alternative codelists.

**Participants.** Comprehensiveness was assessed in a representative CPRD population of 10,966,759 people.

**Results.** After removal of duplicates and GP review, codelists were finalized with 325 unique codes for ethnicity, 558 for LD, 499 for SMI, and 38 for transgender. Compared with comparator codelists, an additional 48,017 (76.6%), 52,953 (68.9%) and 508 (36.9%) people with a LD, SMI or transgender code were identified. The proportions identified for ethnicity, meanwhile, were consistent with expectations for the UK (e.g. 6.1% Asian, 2.7% black, 1.4% mixed).

**Conclusions.** The curated codelists are more sensitive than those widely used in practice and research. Discrepancies between national estimates and primary care records suggest potential record/retention issues. Resolving these requires further investigation and could lead to improved data quality for research.

**STRENGTHS AND LIMITATIONS.**

- This study followed standardised methodology for developing codelists, enhancing the scientific rigour, transparency and reproducibility of the study.
- Codelists were validated against existing codelists, using a representative sample of primary care medical records, comprising >10million patients.
- Only two general practitioners reviewed the codelists, neither of whom were experts in learning disabilities nor severe mental illness. As such, it is possible some conditions were overlooked or under-represented.
- Comparisons with existing codelists were not like-for-like due to differences in population definitions.

For peer review only



For peer review only - http://bmjopen.bmj.com/site/about/guidelines.xhtml

## MAIN TEXT

## 1. INTRODUCTION.

Health inequalities are unfair and avoidable differences in health, which are systematically established and arise from the social conditions in which people are born, grow, live, work and age [1].

In the United Kingdom (UK), addressing health inequalities are a national priority [2]. The National Health Service for England (NHS England) developed 'The CORE20Plus5 Framework', which outlines priority groups and clinical areas requiring accelerated improvement [3]. The 'Core20' refers to the 20% most deprived areas in England, while the 'Plus' refers to population groups that should be identified at a local level, including (but not limited to): ethnic minority groups, people with a learning disability (LD) or autism, people with multiple long-term health conditions, other groups that share protected characteristics (as defined by the Equality Act 2010 [4]), and groups experiencing social exclusion (such as coastal communities, where there may be small areas of high deprivation hidden amongst relative affluence) [3]. The '5' describes key clinical areas requiring accelerated improvement for these populations, namely: maternity, severe mental illness (SMI), chronic respiratory disease, early cancer diagnosis and hypertension case-finding and management [3].

While the CORE20Plus5 framework provides a useful outline of national priorities for health research and clinical practice, there are several ongoing challenges that need to be addressed for researchers to support national strategy [5]. Pertinent among these is the identification and classification of CORE20Plus groups (also referred to as 'socially disadvantaged' or 'marginalised' groups) within electronic health records (EHRs) [5]. EHRs include key administrative clinical data relevant to a person's care, including demographics, progress notes, problems, medications, vital signs, past medical history, immunisations, laboratory data and radiology reports [6]. These data are recorded using clinical coding systems, typically containing hundreds of thousands of concepts [7], which need to be categorised via codelists (i.e. collection of medical terms/codes) to facilitate research.

While methods for codelist development and reporting have previously been published [8–10], and many studies now report the methods used to develop codelists for various medical diagnoses, few papers describe the systematic development of codelists specifically for identifying marginalised populations [9]. Some papers have attempted to develop codelists for certain marginalised groups, including for identifying the autistic population [11], the LGB population [12], and for people who experience multiple long-term conditions [13]. However, there does not appear to be any papers describing the development of codelists for other marginalised groups.

To support future health inequalities research, we set out to develop comprehensive codelists for the identification of four CORE20plus5 groups, namely: people from ethnic minority groups, people with LDs, people with SMI and people who are transgender. We selected these groups because there is a



For peer review only - http://bmjopen.bmj.com/site/about/guidelines.xhtml

general lack of standardised methods for codelist development to describe these inequality populations in EHRs.

## 2. METHOD.

Previously published approaches for codelist development were reviewed [9,10], and Watson et al's [14] approach was deemed the most appropriate because it specifically sets out methods for developing codelists using UK based EHRs.

The approach comprises three steps: **Step 1.** Clearly define the clinical feature of interest a priori; **Step 2.** Assemble a list of codes that may be used to record the clinical feature; **Step 3.** Expert review of codes.

The following presents a detailed overview of the processes undertaken for each step.

### 2.1. **Step 1.** Clearly define the clinical feature of interest a priori.

Watson et al recommend beginning by clearly defining the clinical feature of interest [14]. To do this, they recommend using reliable sources of information, such as the National Institute for Health and Care Excellence (NICE) [15]. For the purposes of this study, we used the following definitions and sources to define marginalised groups of interest:

**Ethnicity.** The Office of National Statistics (ONS) [16] has highlighted that there is no true consensus on what defines an ethnic group, as identification to these are "self-defined and subjectively meaningful to the individual". However, as stated by NHS England, it is generally accepted that ethnicity includes a variety of elements, such as ancestry, culture, identity, religion, language, and physical appearance [17].

We defined ethnicity according to the characteristics set out in the Equality Act 2010, namely: colour, nationality and ethnic or national origins [4]. To this end, UK Census 2021 categories were used to group ethnicity codes (see Table 1) [18].

| Table 1. UK census ethnic categories [15]. |
|---|
| **Asian or Asian British** |
| Indian |
| Pakistani |
| Bangladeshi |
| Chinese |
| Any other Asian background |
| **Black, Black British, Caribbean or African** |
| Caribbean |
| African |
| Any other Black, Black British, or Caribbean background |
| **Mixed or multiple ethnic groups** |
| White and Black Caribbean |
| White and Black African |
| White and Asian |
| Any other Mixed or multiple ethnic background |
| **White** |
| English, Welsh, Scottish, Northern Irish or British |
| Irish |
| Gypsy or Irish Traveller |
| Roma |
| Any other White background |
| **Other ethnic group** |
| Arab |
| Any other ethnic group |

For peer review only

**Learning disability.** The Department of Health and Social Care (DHSC) [19] defines a learning disability as: “a significantly reduced ability to understand new or complex information, to learn new skills (impaired intelligence), with a reduced ability to cope independently (impaired social functioning), which started before adulthood.” According to Public Health England [20]: “a learning disability is different to a learning difficulty, which is a reduced intellectual ability for a specific form of learning and includes conditions such as dyslexia (reading), dyspraxia (affecting physical co-ordination) and attention deficit hyperactivity disorder (ADHD).”

We defined learning disabilities according to the characteristics described by the DHSC. This is because there is significant overlap between a diagnosis of learning disability and learning difficulty, and these two terms have historically been used interchangeably. To capture the most accurate population experiencing inequalities and to avoid possible misclassifications, the more inclusive DHSC definition was used to describe the LD population. This definition for learning disability excludes autism, which is important, as while many people with a learning disability have autism, not everyone with autism has a learning disability. Accordingly, no autism codes were included in the LD codelist.

We did not categorise learning disabilities into groups. They are often described as being ‘mild’, ‘moderate’, or ‘severe’, ‘verbal’ or ‘non-verbal’, and ‘syndromic’ or ‘non-syndromic’, but there is no consensus as to which learning disabilities fall into which categories, and there is individual-level variation within the same disability, making it inappropriate to generalise [21].

**Severe mental illness.** The Diagnostic and Statistical Manual, fifth edition (DSM-V), defines severe mental illness as: “a mental, behavioural, or emotional disorder resulting in serious functional impairment, which substantially interferes with or limits one or more major life activities.” [22].

We defined SMI according to the characteristics described in the DSM-V, and categorised codes according to four recognised conditions, namely: ‘schizophrenia’, ‘bipolar disorder’, ‘severe major depression’ and ‘other psychotic disorders’ [22].

**Transgender.** The UK Government Equality Office [23] defines trans (transgender) people as “People whose gender is different from the gender assigned to them at birth.” They provide the following by way of example: “A trans man is someone that transitioned from woman to man” [23].

We used the definition provided by the Government Equality Office [23]. Most clinical codes do not differentiate between trans men and trans women; therefore, we did not group codes into these categories.

**Patient and public involvement.**

Five members of the public with lived experience of autism, severe mental illness, minoritised ethnicity and/or being transgender contributed to the study. While no one with a learning disability was included,



For peer review only - http://bmjopen.bmj.com/site/about/guidelines.xhtml

a person with autism was able to provide perspective on the differences between autism and learning disability, and their lived experience of the two being conflated with one another.

Initial plans and findings were shared with these collaborators during quarterly meetings. During these meetings, collaborators provided feedback on the codelists, the language used to define them, and how to handle outdated or offensive terminology (such terms were retained within the codelist to ensure completeness but were omitted from the manuscript, so as not to perpetuate their use in medical literature.

### 2.2. **Step 2.** Assemble list of codes that may be used to record the clinical feature.

In the second step, an online thesaurus was used to develop a list of synonyms associated with the clinical features of interest. An overview of the synonyms used for each clinical feature is presented in Table 2.

**Table 2.** List of synonyms used for each clinical feature

| Clinical feature | Synonyms |
|---|---|
| **Ethnicity** | Nationality, Nationalities, Nation(s), Race(s), Racial, Colour, Ethnic, Ethnicity, Minority, Minorities |
| **Learning Disability** | Intellectual disability, Intellectual disabilities, Learning disability, Learning Disabilities, ID(s), LD(s) |
| **Severe mental illness** | Serious mental illness, Serious mental illnesses, Severe mental illness, Severe mental illnesses, SMI(s), Schiz*, Psychosis, Bipolar, Major depress* |
| **Transgender** | Trans, Transsexual, Transgender, Transgender, Gender dysmorphia, Trans man, Trans woman, Trans men, Trans women, Transfeminine, Transmasculine, Gender Surgery, LGBT. |

These identified synonyms were used to search literature and databases to find relevant published codelists related to the inequality groups. One major resource used was the October 2023 Clinical Practice Research Datalink (CPRD) Bibliography [24]. CPRD is one of the largest research databases of electronic, anonymised, and longitudinal medical records from primary care in the world [25]. The CPRD Bibliography is a resource which provides a record of all published studies (>3,000 as of October 2023) using CPRD data, and this aided in our search for publications. The relevant publications were identified using our list of synonyms and codelists used in these publications were subsequently downloaded, where they had been made available.

To capture codelists used in non-CPRD studies, we also searched for, and downloaded, relevant codelists from four widely used codelist repositories, namely: OpenCodelists [26], the Health Data



For peer review only - http://bmjopen.bmj.com/site/about/guidelines.xhtml

Research UK (HDRUK) Phenotype Library [27], the University of Cambridge Codelist Repository [28] and the London School of Hygiene and Tropical Medicine (LSHTM) Data Compass [29].

Once all relevant published codelists were identified, they were combined into one excel file for each inequality group. This gave us four files: one for ethnicity, LD, SMI, and transgender. Within each of these files, any duplicate codes were removed, and remaining codes grouped by the two main coding systems used in the UK: Read and systematized nomenclature of medicine clinical terminology (SNOMED-CT). Read was generally used in primary care until 2018, and SNOMED-CT thereafter, following the enablement of SNOMED-CT within Primary care systems [30]. This provided an aggregated list encompassing every code (excluding duplicates) that has been used and published either through CPRD literature or through an online codelist database.

These aggregated codelists were then checked for accuracy using the CPRD Code Browser, which is a tool specific to CPRD and is provided to researchers who gain access to their data. This tool contains the diagnostic description, the alphanumeric Read / SNOMED-CT code originally used by the general practitioner (GP) to enter the clinical information, and CPRD's proprietary 'medcode' / 'medcodeid' (which is simply a numeric equivalent of the Read / SNOMED-CT code) [30]. The tool allowed for easy browsing by either using a known code or description to check code accuracy. Searching by the medical descriptors for the code, or "terms," was useful because each search provided a list of all possible codes. For example, a search for "psychosis" showed every diagnostic term and associated code available from primary care GP records. This was useful to cross reference each of the individual codes and ensure all relevant options have been included. In line with the method described by Watson et al [14], inaccurate codes were removed or corrected, and additional codes (not previously identified) added if they were associated with the diagnostic definition of one or more eligible codes.

### 2.3. **Step 3.** Expert review of codes.

In the final step, the codelists were sent to a GP (IVH) for review. The codes were scored using the following three-point scale:

1. Definitely include: the code accurately defines the clinical feature of interest, and GPs would use it.
2. Uncertain: it remains unclear whether the code accurately reflects the clinical feature of interest, or whether GPs would use it.
3. Definitely exclude: the code does not define the clinical feature of interest, and GPs would avoid.

Codes assigned a score of 2 ('Uncertain') or 3 ('Definitely exclude') were reviewed by a second GP (BN). All codes assigned a score of 3 ('Definitely exclude') by both reviewers were removed from the codelists. All codes receiving a score of 2 from either reviewer were retained as 'uncertainty variables',



For peer review only - http://bmjopen.bmj.com/site/about/guidelines.xhtml

which are recommended for sensitivity analyses [14]. These variables are typically less frequently used by GPs, but may identify additional, potentially relevant, cases [14].

### 2.4. Assessing codelist comprehensiveness

To explore the comprehensiveness of the developed codelists, we used the CPRD database to ascertain the number of people with at least one recording of an included code for each list. We restricted the population to those who had been registered with their CPRD Aurum practice since at least 1st January 2019 and who had neither died nor deregistered from their practice by August 2022 (the date of last data collection).

In the context of CPRD studies, researchers have been able to acquire already categorised ethnicity data through linkage with the Hospital Episode Statistic (HES) [31]. Derived ethnicity data is also now available through CPRD at an additional cost [32]. The ethnicity codelist we generated will service to those researchers who are using other non-CPRD datasets, or ones who may not have access to either the linked HES data or the CPRD derived ethnicity.

For the other variables, validated codelists from other sources were selected as comparisons, seeking to match the scope of each list as closely as possible. Codelists for LD and SMI were sourced from the Quality and Outcomes Framework (QOF: the pay for performance scheme for general practices in the UK) [33]. QOF includes incentives for practices to keep a register of patients with certain conditions, including LD and SMI, and produces validated codelists for this purpose. We used lists from the QOF Business Rules version 45; “Learning Disability codes” (list name LD_COD) and "Psychosis and schizophrenia and bipolar affective disease codes" (list MH_COD) as our comparison lists. For the transgender category, no QOF list exists, and literature is slim. We identified two identical previously published codelists that were used to compare to our newly developed codelist.

## 3. RESULTS.

A review of 87 relevant publications and additional codelist repositories identified a total of 55 codelists: 16 for ethnicity, 16 for LD, 21 for SMI and 2 for transgender. After removing duplicates and expert review, 388 codes were excluded, and 1,420 total unique codes remained: 325 codes for ethnicity, 558 codes for LD, 499 codes for SMI and 38 codes for transgender. The full review process is demonstrated in figure 1.

(Figure 1 Here)

The codes were run in the source population of CPRD (n=10,966,759) to ascertain the number of people captured by the codelists (see Table 3). People were identified by the presence of one or more codes related to the inequality group of interest. Ethnicity data were available for 4,384,017 (40.0%) individuals. Of these, 3,112,319 (71.0%) were ethnically white; 285,126 (6.50%) were ethnically Asian;



For peer review only - http://bmjopen.bmj.com/site/about/guidelines.xhtml

116,570 (2.66%) were ethnically black; 63,024 (1.44%) were of mixed ethnicity; 94,693 (2.16%) were categorised as ‘other;’ and 712,286 (16.3%) were of unknown ethnicity.

110,692 people were found to have LD (1.00%), 129,788 people had SMI (1.18%) and 1,884 people were transgender (0.02%). This represented an increased capture of 48,017 (76.6%) for LD, 52,953 (68.9%) for SMI, and 508 (36.9%) for transgender (see Table 4).

For peer review only

**Table 3.** Descriptives for CPRD population, including frequencies between ethnic groups derived from our curated codelist.

| Population Total (N) | Mean age (SD*) | Age range | Frequency males (%) | Frequency females (%) |
|---|---|---|---|---|
| 10,966,759 | 43.3 (22.8) | 4 – 100 years old | 5,554,024 (50.6) | 5,412,735 (49.4) |

| Ethnicity | Frequency from curated codelist (N) | Frequency percent (%) |
|---|---|---|
| **White** | 3,112,319 | 71.0 |
| **Asian or Asian British** | 285,126 | 6.50 |
| **Black, Black British, Caribbean or African** | 116,570 | 2.66 |
| **Mixed or multiple ethnic groups** | 63,024 | 1.44 |
| **Other ethnic group** | 94,693 | 2.16 |
| **Unknown** | 712,286 | 16.3 |

*SD = Standard deviation

**Table 4.** Frequencies of populations extracted from CPRD, comparing original and curated codelist for LD, SMI and transgender groups.

| Inequality Group | Original Codelist (N) | Curated Codelist (N) | Difference in Identification (N) | Percent Increase (%) |
|---|---|---|---|---|
| **Learning disability** | 62,675 | 110,692 | 48,017 | 76.6 |
| **Severe mental illness** | 76,835 | 129,788 | 52,953 | 68.9 |
| **Transgender** | 1,376 | 1,884 | 508 | 36.9 |



For peer review only - http://bmjopen.bmj.com/site/about/guidelines.xhtml

## 4. DISCUSSION.

### 4.1. Summary.

We created comprehensive codelists for identifying marginalised groups of interest including ethnicity, LD, SMI and transgender records in EHRs. Compared with the commonly used alternatives, our codelists improved data capture by 76.6% for LD, 68.9% for SMI and 36.9% for transgender records. These codelists have been made publicly available for others to use in their own research: https://osf.io/8skze/ [34]. They are readily available for use in UK research, but need to be adapted for international research, where different code systems are used. We provide code descriptions, alongside codes, to help facilitate this process.

### 4.2. Strengths and limitations.

This study has several strengths. First, codelists were identified through multiple sources, including CPRD publications and code list repositories, maximising the chances of identifying potentially relevant codes. Second, instead of accepting codes at face value, an expert review was implemented, with potentially irrelevant codes removed or segregated as uncertainty variables. The segregated codes will aid researchers for use in sensitivity analyses, if they find it relevant to their projects.

This study also has a number of limitations. First, it only used codes from code lists arising from CPRD publications, or uploaded to code list repositories, and, despite several safety netting procedures, it is possible not all relevant codes were captured. This is exemplified by the fact that only 29% of relevant publications made their code lists publicly available, meaning there is potential for some codes to be missed. Second, while we compared the comprehensiveness of our codelists against commonly used alternatives (e.g. QOF), the comparisons were not like-for-like, due to differences in population definitions. For example, the QOF definition of SMI does not include severe major depression, and these differences may partially explain some of the increase observed in our codelist. Finally, only two experts reviewed the codelists, both of whom were general practitioners. While a strength in terms of them being representative of the population responsible for entering medical codes in primary care clinical systems, they may be less familiar with the plethora of medical diagnoses associated with some conditions such as SMI and LD, which may have resulted in some conditions being omitted from our codelists. We encourage experts (e.g. learning disability nurse) to feedback and refine these lists.

### 4.3. Comparison with previously published literature.

This research builds on the extant literature by synthesising the efforts of those who have previously curated code lists for the identification of marginalised groups. For example, Boyd et al [35] previously created a code list for identifying people who are transgender, which included a total of eight Read codes. Our list includes an additional 30 Read codes (along with the corresponding SNOMET-CT codes), enabling higher resolution and identification of people who are transgender.

### 4.4. Implications for research.

The findings of our study have several implications for research. Since only a small proportion of researchers made their codelists publicly available, there is a need to encourage researchers to share their codelists. This will expedite inequalities research and prevent duplication of work. Second, while the percentages of ethnicity found in the population closely mirrored the frequencies reported in the UK census 2021 [18], we found that other marginalised groups were under-represented in the CPRD dataset (1.00%, 1.18% and 0.02% were recorded as having a LD, SMI, or transgender code, respectively, compared with national estimates of 2.16%, 2.00-3.00% and 0.20% respectively [36–38]). Further research is needed to understand why these frequencies do not match national estimates.

The implementation of our codelists in inequalities research will serve to simplify the complex process of identifying these groups of interest. The codelists will improve the extraction and management of diverse health information obtained from primary health care records, thus providing researchers with flexible tools that can be adapted to identify marginalised populations in accordance with local definitions. This will boost the efficiency at which inequalities research is conducted using EHR.

## 5. CONCLUSION.

The outputs from our study provide an important contribution to future healthcare research since it is tackling the topic of addressing inequalities, which is held central to the NHS Long Term Plan for this decade [2]. With the combination of this work and previously published work developing codelists for marginalised populations [11–13], this will improve the representation of inequality groups in future research using EHR. We encourage others to use and edit our codelists as they see fit, and to follow our methods to develop comprehensive codelists for the identification of other groups that may be of interest to their research topics.

## REFERENCES.

1 What are healthcare inequalities? NHS Engl. 2021. https://www.england.nhs.uk/about/equality/equality-hub/national-healthcare-inequalities-improvement-programme/what-are-healthcare-inequalities/ (accessed 28 August 2024)

2 NHS Long Term Plan. NHS Engl. 2020. https://www.longtermplan.nhs.uk/ (accessed 4 September 2024)

3 Core20PLUS5 (adults) – an approach to reducing healthcare inequalities. NHS Engl. Digit. 2023. https://www.england.nhs.uk/about/equality/equality-hub/national-healthcare-inequalities-improvement-programme/core20plus5/ (accessed 28 August 2024)

4 Equality Act 2010. UK Gov. Legis. 2010. https://www.legislation.gov.uk/ukpga/2010/15/contents (accessed 4 September 2024)

5 Whitaker KL, Perchyk T, Kerrison RS, *et al.* Challenges in understanding inequities in help-seeking for possible cancer symptoms. *BMC Glob Public Health*. 2024;2:49. doi: 10.1186/s44263-024-00082-1

6 Electronic Health Records. Cent. Medicare Medicaid Serv. 2024. https://www.cms.gov/priorities/key-initiatives/e-health/records (accessed 4 September 2024)



For peer review only - http://bmjopen.bmj.com/site/about/guidelines.xhtml

7 Bhattacharyya S. *Introduction to SNOMED CT*. Singapore: Springer 2016.

8 Benchimol EI, Smeeth L, Guttmann A, *et al.* The REporting of studies Conducted using Observational Routinely-collected health Data (RECORD) statement. *PLoS Med*. 2015;12:e1001885. doi: 10.1371/journal.pmed.1001885

9 Matthewman J, Andresen K, Suffel A, *et al.* Checklist and guidance on creating codelists for electronic health records research. *NIHR Open Res*. 2024;4:20. doi: 10.3310/nihropenres.13550.1

10 Williams R, Kontopantelis E, Buchan I, *et al.* Clinical code set engineering for reusing EHR data for research: A review. *J Biomed Inform*. 2017;70:1–13. doi: 10.1016/j.jbi.2017.04.010

11 Hagberg KW, Jick SS. Validation of autism spectrum disorder diagnoses recorded in the Clinical Practice Research Datalink, 1990-2014. *Clin Epidemiol*. 2017;9:475–82. doi: 10.2147/CLEP.S139107

12 Kronk C, Tran GQ, Wu DTY. Creating a Queer Ontology: The Gender, Sex, and Sexual Orientation (GSSO) Ontology. *Stud Health Technol Inform*. 2019;264:208–12. doi: 10.3233/SHTI190213

13 Kuan V, Denaxas S, Patalay P, *et al.* Identifying and visualising multimorbidity and comorbidity patterns in patients in the English National Health Service: a population-based study. *Lancet Digit Health*. 2023;5:e16–27. doi: 10.1016/S2589-7500(22)00187-X

14 Watson J, Nicholson BD, Hamilton W, *et al.* Identifying clinical features in primary care electronic health record studies: methods for codelist development. *BMJ Open*. 2017;7:e019637. doi: 10.1136/bmjopen-2017-019637

15 NICE guidelines. Natl. Inst. Health Care Excell. 2024. https://www.nice.org.uk/about/what-we-do/our-programmes/nice-guidance/nice-guidelines (accessed 29 August 2024)

16 Ethnic group, national identity and religion. Off. Natl. Stat. 2021. https://www.ons.gov.uk/methodology/classificationsandstandards/measuringequality/ethnicgroupnationalidentityandreligion (accessed 4 September 2024)

17 Ethnicity. NHS Engl. Digit. 2022. https://digital.nhs.uk/data-and-information/data-collections-and-data-sets/data-sets/mental-health-services-data-set/submit-data/data-quality-of-protected-characteristics-and-other-vulnerable-groups/ethnicity (accessed 4 September 2024)

18 Ethnic group, England and Wales. Off. Natl. Stat. 2022. https://www.ons.gov.uk/peoplepopulationandcommunity/culturalidentity/ethnicity/bulletins/ethnicgroupenglandandwales/census2021 (accessed 30 August 2024)

19 Valuing People - A New Strategy for Learning Disability for the 21st Century. Dep. Health Soc. Care. 2001. https://www.gov.uk/government/publications/valuing-people-a-new-strategy-for-learning-disability-for-the-21st-century (accessed 4 September 2024)

20 Learning disability - applying All Our Health. Public Health Engl. 2023. https://www.gov.uk/government/publications/learning-disability-applying-all-our-health/learning-disabilities-applying-all-our-health (accessed 4 September 2024)

21 Karmiloff-Smith A, Al-Janabi T, D'Souza H, *et al.* The importance of understanding individual differences in Down syndrome. *F1000Research*. 2016;5:F1000 Faculty Rev-389. doi: 10.12688/f1000research.7506.1

22 Diagnostic and Statistical Manual of Mental Disorders (DSM-5-TR). Am. Psychiatr. Assoc. 2024. https://www.psychiatry.org:443/psychiatrists/practice/dsm (accessed 4 September 2024)


For peer review only



For peer review only - http://bmjopen.bmj.com/site/about/guidelines.xhtml

23 Trans people in the UK. Gov. Equal. Off. 2018. chrome-extension://efaidnbmnnnibpcajpcglclefindmkaj/https://assets.publishing.service.gov.uk/media/5b3a478240f0b64603fc181b/GEO-LGBT-factsheet.pdf (accessed 29 August 2024)

24 Bibliography. CPRD. 2024. https://www.cprd.com/bibliography (accessed 4 September 2024)

25 Clinical Practice Research Datalink. CPRD. 2024. https://www.cprd.com/ (accessed 4 September 2024)

26 OpenCodelists. OpenCodelists. 2022. https://www.opencodelists.org/ (accessed 28 August 2024)

27 HDRUK Phenotype Library. HDRUK Phenotype Libr. 2024. http://phenotypes.healthdatagateway.org/ (accessed 28 August 2024)

28 Department of Public Health and Primary Care. Univ. Camb. 2024. https://www.phpc.cam.ac.uk/pcu/research/researchgroups/crmh/cprd_cam/codelists/v11/ (accessed 28 August 2024)

29 Code list. LSHTM Data Compass. 2024. https://datacompass.lshtm.ac.uk/view/keywords/Code_list.html (accessed 4 September 2024)

30 Clinical coding – SNOMED CT. NHS Engl. 2023. https://www.england.nhs.uk/long-read/clinical-coding-snomed-ct/ (accessed 4 September 2024)

31 Hospital Episode Statistics (HES). NHS Engl. Digit. 2024. https://digital.nhs.uk/data-and-information/data-tools-and-services/data-services/hospital-episode-statistics (accessed 4 September 2024)

32 CPRD algorithm derived data. CPRD. 2024. https://www.cprd.com/cprd-algorithm-derived-data (accessed 30 August 2024)

33 Quality and Outcomes Framework (QOF). NHS Engl. Digit. 2023. https://digital.nhs.uk/data-and-information/data-tools-and-services/data-services/general-practice-data-hub/quality-outcomes-framework-qof (accessed 29 August 2024)

[dataset] 34 Kerrison, RS. Code lists for inequalities research. Open Sci. Framew. 2024. https://doi.org/10.17605/OSF.IO/8SKZE (accessed 6 April 2025)

35 Boyd I, Hackett T, Bewley S. Care of Transgender Patients: A General Practice Quality Improvement Approach. *Healthc Basel Switz*. 2022;10:121. doi: 10.3390/healthcare10010121

36 How common is learning disability? Mencap. 2024. https://www.mencap.org.uk/learning-disability-explained/research-and-statistics/how-common-learning-disability (accessed 4 September 2024)

37 Mental health facts and statistics. Mind. 2020. https://www.mind.org.uk/information-support/types-of-mental-health-problems/mental-health-facts-and-statistics/ (accessed 4 September 2024)

38 Gender identity, England and Wales - Office for National Statistics: Census 2021. Off. Natl. Stat. 2023. https://www.ons.gov.uk/peoplepopulationandcommunity/culturalidentity/genderidentity/bulletins/genderidentityenglandandwales/census2021 (accessed 3 December 2024)



For peer review only - http://bmjopen.bmj.com/site/about/guidelines.xhtml

**Figure 1.** Process outlining codelist review and development.

For peer review only



For peer review only - http://bmjopen.bmj.com/site/about/guidelines.xhtml

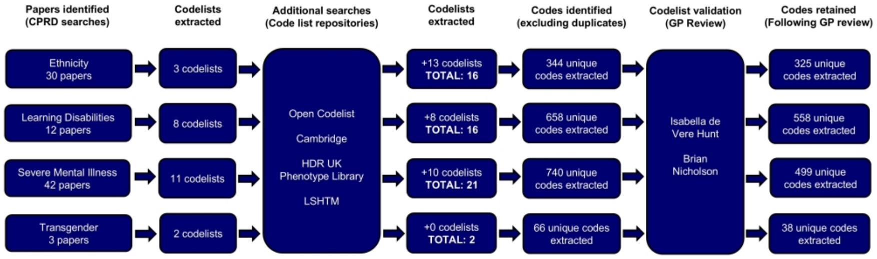


Caption : Figure 1. Process outlining codelist review and development.

338x190mm (300 x 300 DPI)
